# Supplementary material for: Slx5/Slx8‐dependent ubiquitin hotspots on chromatin contribute to stress tolerance
Source: EMBO J. 2019 Apr 23;38(11):e100368. doi: 10.15252/embj.2018100368 (PMC6545562; doi:10.15252/embj.2018100368)
Supplement: Supplementary file 1 — Appendix [file EMBJ-38-e100368-s001.pdf]

APPENDIX MATERIALS

Contents

Appendix Figures S1-S12 and Appendix Figure Legends

Appendix Supplementary Methods

Appendix Tables S1-3

Appendix References

Appendix Figure S1.

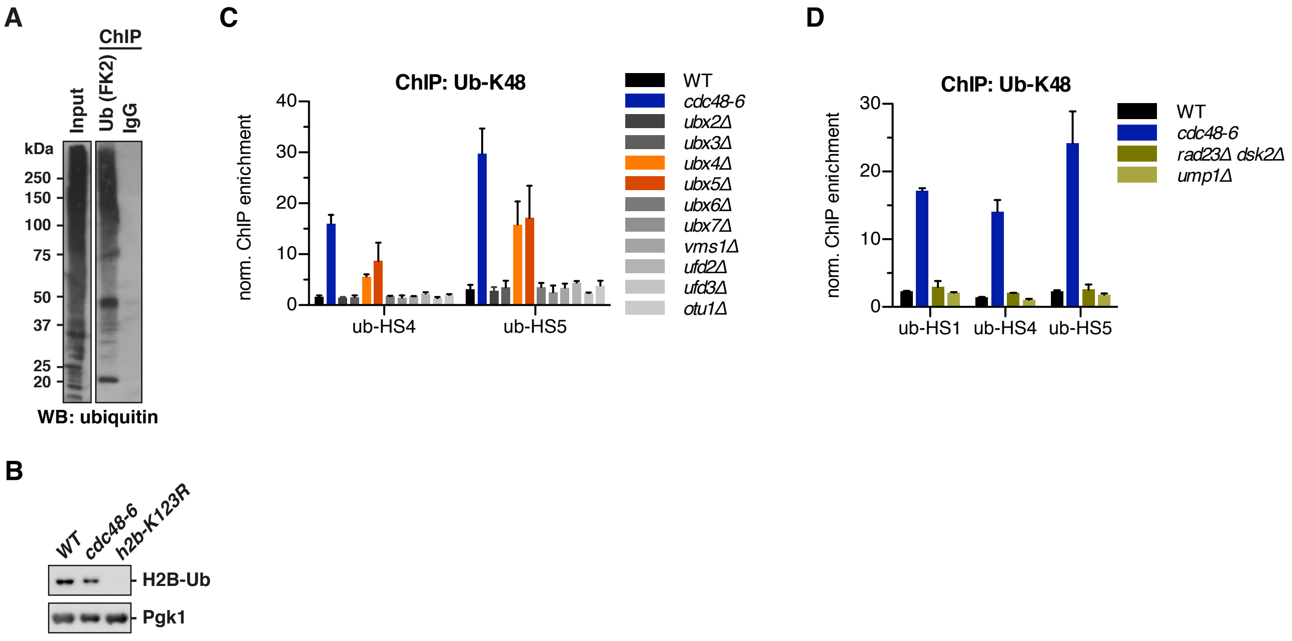

Appendix Fig. S1, related to Fig. 1

(A) The ubiquitin (FK2) antibody efficiently enriches ubiquitin-conjugates in ChIP-experiments. Material from ubiquitn (FK2) and non-specific IgG-control ChIP experiments was analyzed by Western blot (WB) using the P4D1-ubiquitin antibody.

(B) Mutation of histone H2B lysine 123 to arginine (*h2b-K231R*) leads to loss of H2B ubiquitylation. Levels of H2B ubiquitylation are mildly decreased in a *cdc48-6* strain. WB for Pgk1 served as control.

(C) Ubx4 and Ubx5 are required for efficient removal of ubiquitylated proteins from ub-HS sites. Ub-K48 ChIP-qPCR data show an increase at ub-HSs in *ubx4Δ* and *ubx5Δ*, but not in the other tested Cdc48-cofactor mutants. Note that we used *ubx5Δ* strains in some experiments to enhance ubiquitin ChIP signals while avoiding the more detrimental *cdc48-3* or *cdc48-6* alleles. Data represent means ± SD (*n* = 3).

(D) Impairment of the targeting of Cdc48 substrates to the proteasome (*rad23Δ dsk2Δ*) or deletion of a proteasome assembly chaperone (*ump1Δ*) does not cause accumulation of ubiquitylated substrates at ub-HS sites. ub-K48 ChIP-qPCR was performed with the indicated strains. Data represent means ± SD (*n* = 2).

Appendix Figure S2.

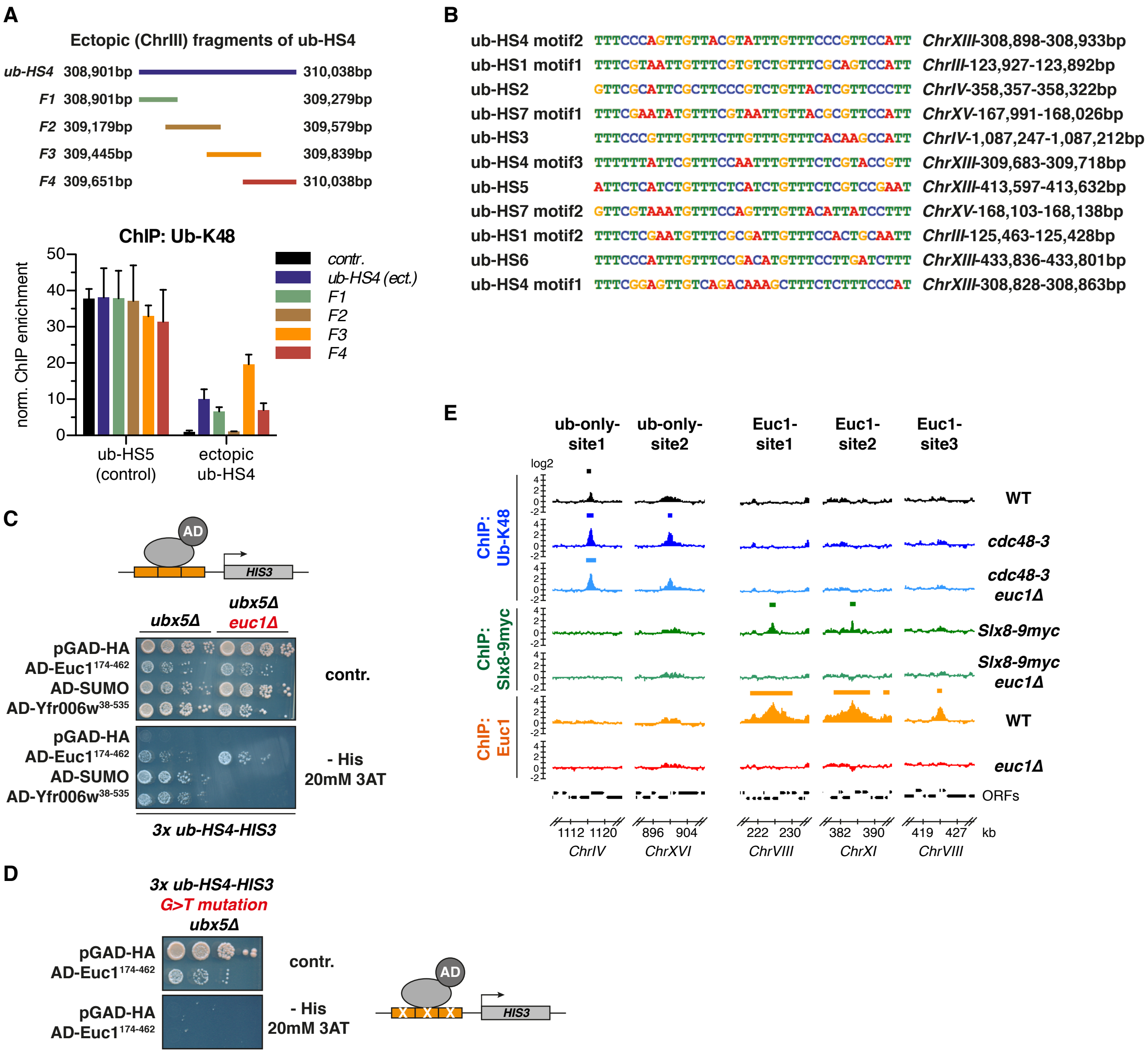

Appendix Fig. S2, related to Fig. 2

(A) Fragments of ub-HS4 as indicated were ectopically integrated at the *LEU2*-locus (see Fig. 2A) and ChIP for K48-linked ubiquitin chains was performed. qPCR was used to quantify enriched DNA at the endogenous ub-HS5 and the ectopic ub-HS4 fragments using primers designed to bind the YIplac128 backbone at a position close to the cloned ub-HS4 fragment. contr.: control, empty YIplac128-vector was integrated at *LEU2*. Experiments were performed in strains with the *cdc48-6* allele. Note that we used truncations of F1 (containing ub-HS4-motif2, (B)) for further fine-mapping in Fig. 2B, while F3 and F4 also give an ectopic ub-HS because they harbor another ub-HS-motif (ub-HS4-motif3, (B)). Data represent means ± SD ($n = 3$).

(B) Alignment of the ub-HS-motifs identified by MEME (Fig. 2C). 1–2 kb regions around the main ub-peaks were used as input sequences for the MEME-online tool to predict sequence motifs. Note that several ub-HS-motifs occur in some of the ub-HS sites, see also (A).

(C) Binding of AD-SUMO and AD-Yfr006w$^{38\text{-}535}$ depends on endogenous Euc1. The Y1H reporter strain (*ubx5Δ, 3x ub-HS4-HIS3*) and an isogenic *euc1Δ* strain were transformed with plasmids isolated in the Y1H screens. We note that secondary structure predictions suggest a transmembrane domain in the N-terminal region of Yfr006w that is missing from the Y1H hits, possibly leading to aberrant protein localization and/or interaction with Euc1. Serial dilutions of cells were spotted on control or selective media lacking histidine with 20 mM 3-amino-triazole (3AT) added to suppress growth by background activation of *HIS3*. Cells were grown at 30°C for 3 days (contr.) or 4 days (-His 20mM 3AT).

(D) Euc1 does not bind to the mutated ub-HS4-motif in a Y1H assay. We introduced the G>T mutation of ub-HS4 F7 described in Fig. EV1F in the three copies of the ub-HS4-motif upstream of *HIS3* in a Y1H reporter strain. AD-Euc1$^{174\text{-}462}$ did not activate *HIS3* in this strain (compare to (C)). Experiment was performed as described in (C).

(E) Summary of ChIP-chip data for sites with major ubiquitin-enrichment in *cdc48* mutants (*cdc48-3*) without Slx8 and Euc1 enrichment (ub-only-sites, left panels) and sites with major Euc1 enrichment, two of which also show Slx8 accumulation, but no ubiquitin signals (Euc1-sites, right panels). ChIP-chip experiments were performed as described in Fig. 1A-B and Fig. 2H.

We note that, in agreement with a previous study (van de Pasch *et al*, 2013), we did not see any binding of Slx8-9myc to centromeres in ChIP-chip or ChIP-qPCR. In contrast to that study, however, we also did not detect any Slx5 enrichments at centromeres when probed by qPCR for 3HA-Slx5 or endogenous Slx5. The discrepancies might be due to the use of a non-functional *SLX5*-allele by van de Pasch *et al* (see statement in their methods section).

Appendix Figure S3.

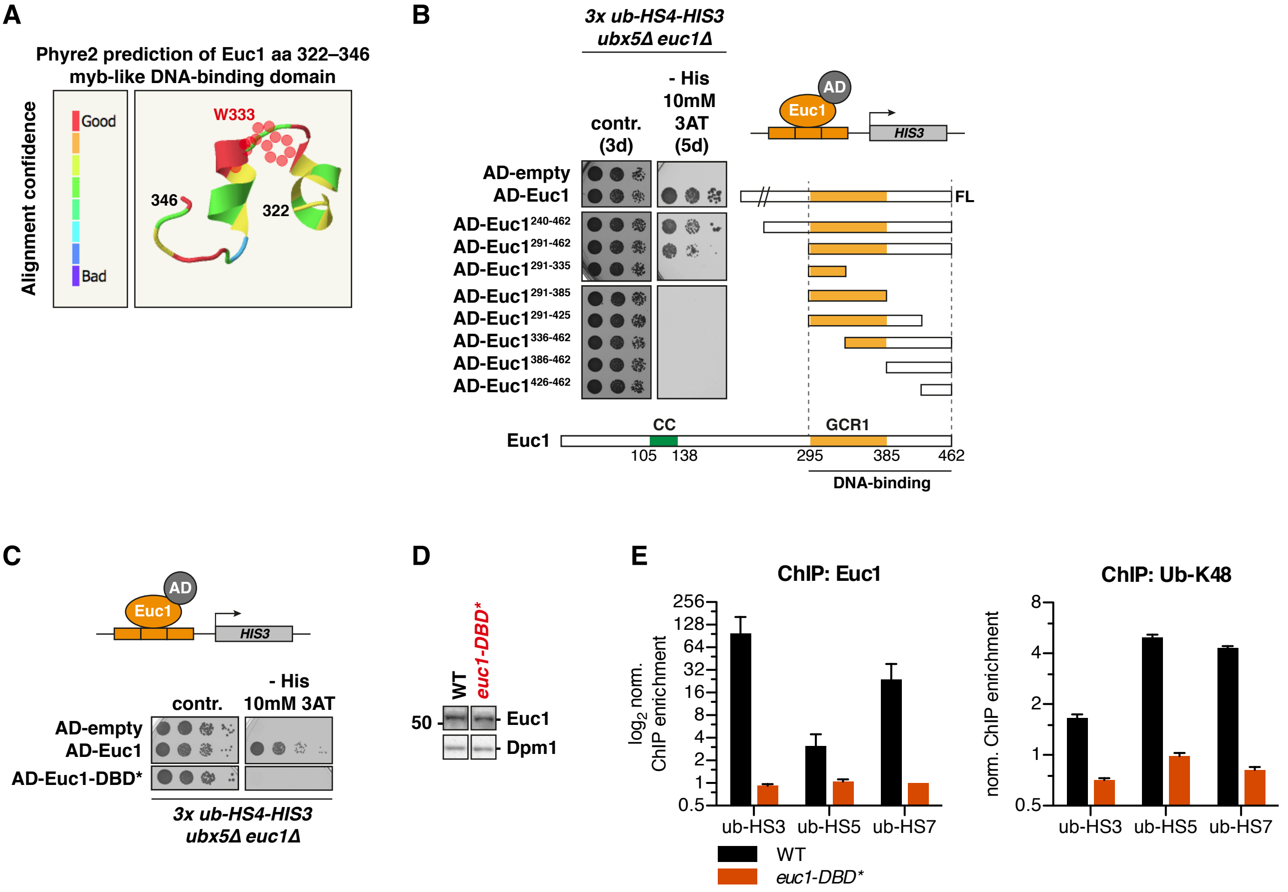

Appendix Fig. S3, related to Fig. 2

(A) Phyre2 structural prediction result (Kelley *et al*, 2015) for part of the Euc1 GCR1 domain (aa 322–346) that shows homology to the DNA/RNA-binding 3-helical bundle fold of the myb-like DNA-binding domains (DBD) of the homeodomain family. Structural prediction is color-coded for the alignment confidence with PDB-entry d1x58a1 (*Mus musculus* Terb1). A conserved tryptophan (W333) predicted to display strong mutational sensitivity is highlighted in red.

(B) The GCR1 domain and C-terminus of Euc1 are required and sufficient for DNA binding in Y1H assays. Y1H assays as described in Fig. 2E were performed to map the DNA-binding domain of Euc1. The AD-Euc1$^{291\text{-}462}$ fragment is sufficient to bind DNA in the Y1H assay, however truncations from either the C- or N-terminus lead to loss of DNA binding.

(C) Mutation of the myb-like DNA-binding domain leads to loss of Euc1 binding to the ub-HS-motif in Y1H assays. The W333A, R334A-mutation (hereafter *euc1-DBD**) led to a complete loss of Euc1 DNA binding. Assay was performed as in Fig. 2E, cells were grown for 3 days at 30°C.

(D) Expression levels of Euc1 in WT and *euc1-DBD** strains used in (E) as probed by WB. Euc1 was probed with the Euc1-antibody and Dpm1 levels served as loading control. Sections were cropped from the same exposure.

(E) The Euc1-DBD is required for Euc1 binding and ubiquitin enrichment at endogenous ub-HS sites. ChIP-qPCR using an Euc1-specific or ub-K48 antibody was performed in either a WT strain or a strain expressing the *euc1-DBD** allele (W333A, R334A). Data represent means ± SD (*n* = 2).

Appendix Figure S4.

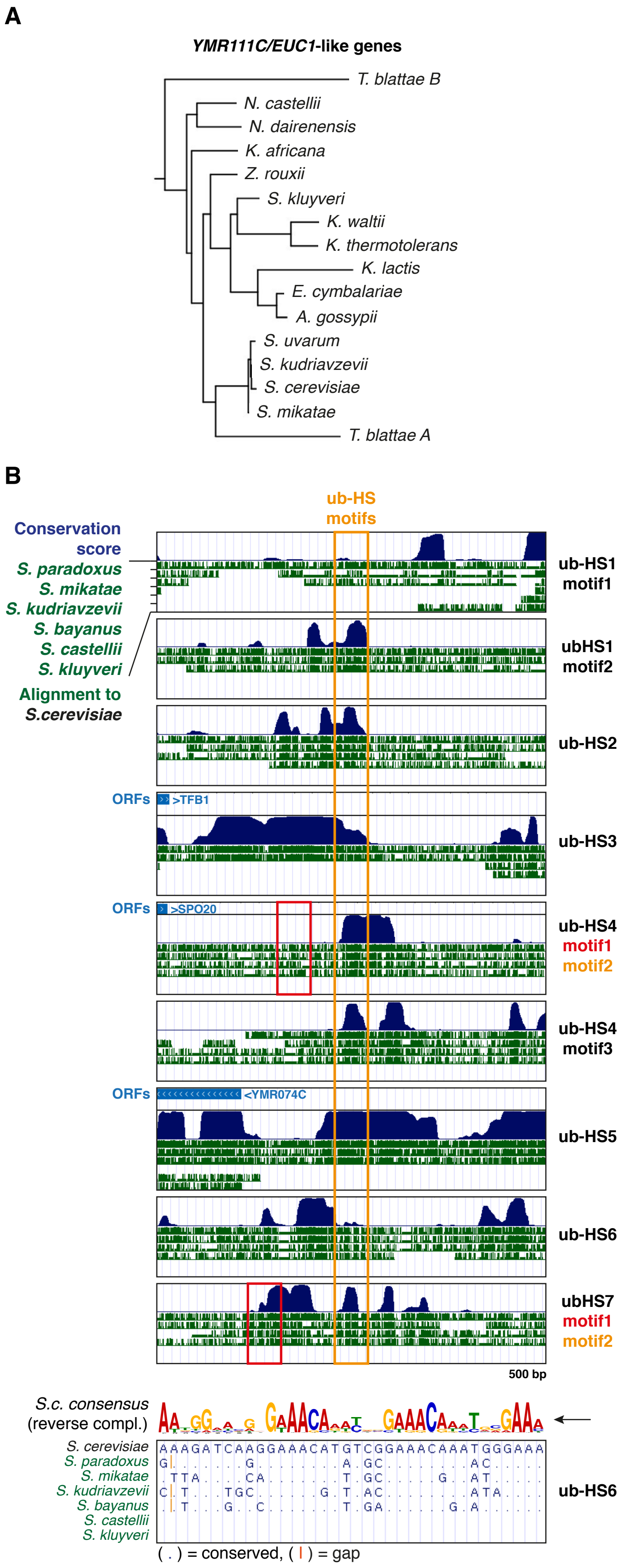

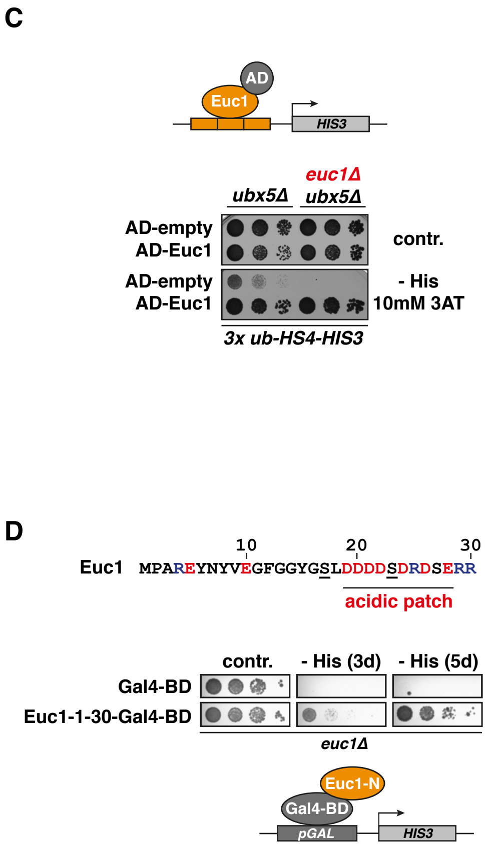

Appendix Fig. S4, related to Fig. 3

(A) Phylogenetic tree of *YMR111C/EUC1*-like genes. The Yeast Gene Order Browser was used to identify orthologs of *YMR111C/EUC1* in other yeasts and to generate a phylogenetic tree with the MUSCLE algorithm and PhyML (http://ygob.ucd.ie/ (Byrne & Wolfe, 2005)).

(B) Ub-hotspot sequences are partially conserved in closely related *Saccharomyces* species. The 7 yeast Multiz Alignment & Conservation tool of the UCSC Genome Browser was used to retrieve alignments of sequences corresponding to ub-HS-motifs from other *Saccharomyces* species. Top: 500 bp stretches are displayed, with the site corresponding to the ub-HS-motif marked in orange. Bottom: Alignment of ub-HS6, which has an overall poor conservation score, reveals at base-pair resolution that residues important in the *S. cerevisiae* consensus appear conserved in other *Saccharomyces* species.

(C) Endogenous Euc1 acts as a TF in reporter-gene assays. The Y1H reporter strain described in Fig. 2D (*3x ub-HS4-HIS3 ubx5Δ*) or the same strain with *EUC1* deleted (*euc1Δ*) was transformed with the indicated plasmids and a growth assay was performed as described in Fig. 2E. Note that endogenous Euc1 weakly drives activation of the *HIS3* reporter gene. Cells were grown at 30°C for 5 days.

(D) The Euc1 N-terminal 30 amino acids harbor an acidic patch and are sufficient for transactivation. Top: Euc1 aa 1-30 are shown with negatively charged residues shaded in red and positively charged residues in blue. Underlined residues have been annotated to be phosphorylated in UniProt. Bottom: the Gal4-BD was C-terminally fused to Euc1 aa 1–30 and the activation of a *GAL1*-promoter-controlled *HIS3* reporter-gene was monitored. The PJ69-7a Y2H strain with *EUC1* deleted was used.

Appendix Figure S5.

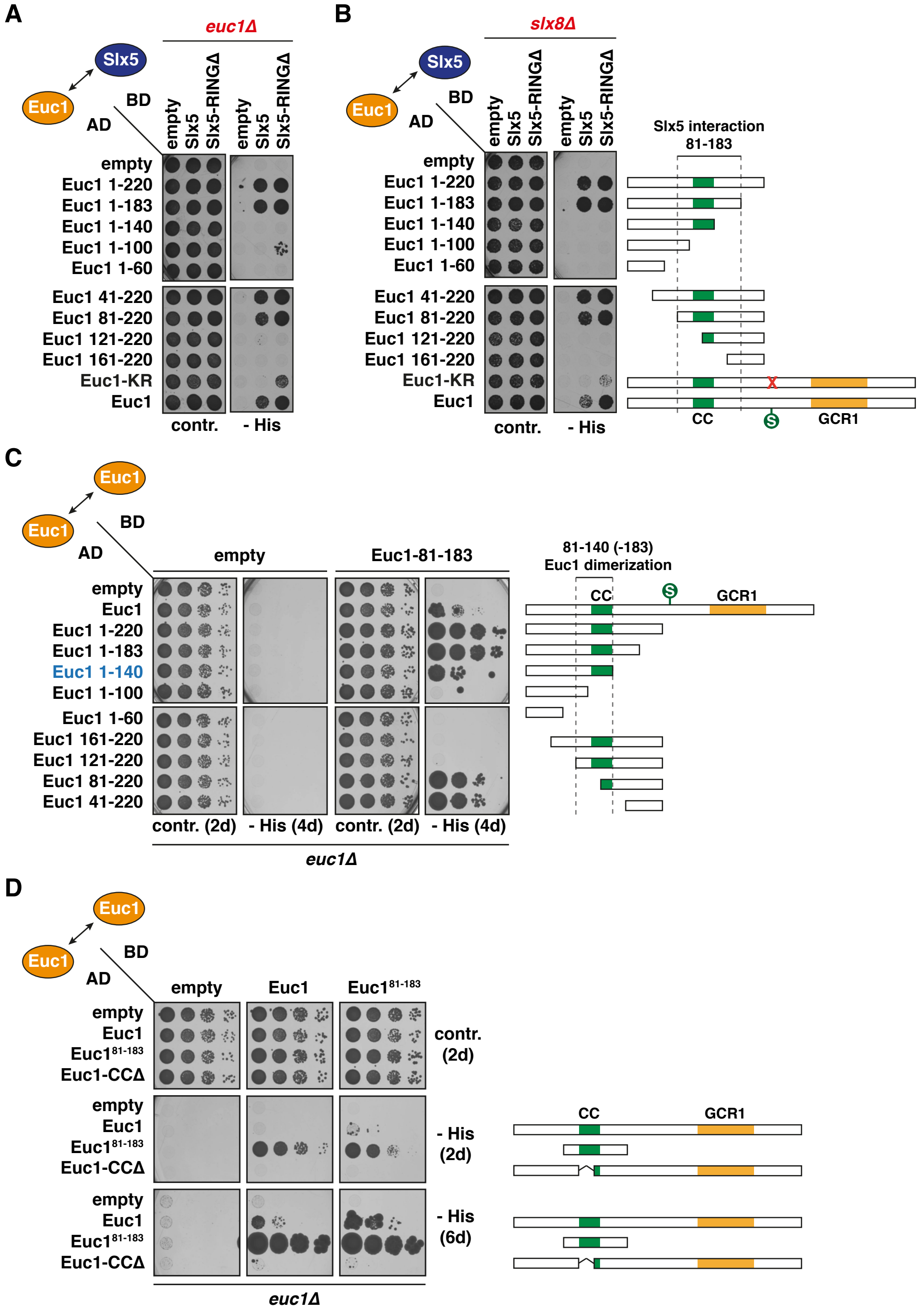

Appendix Fig. S5, related to Fig. 5

(A–B) Endogenous Euc1 and Slx8 are not required for the Euc1–Slx5 interaction in Y2H. Experiment was performed as in Fig. EV3B in an *euc1Δ* (A) or an *slx8Δ* genetic background (B). Cells were grown at 30°C for 2 days (A) or 3 days (B).

(C) The minimal region of Euc1 required for dimerization maps to aa 81–140. Y2H assay to map the stretch required for dimerization of Euc1 around the CC domain. To avoid artifacts by interactions with endogenous Euc1, we used an *euc1Δ* strain. Note that the region between aa 140–183 likely also contributes to dimerization. Serial dilutions were spotted and cells were grown at 30°C for 2 (control) or 4 days (- His).

(D) The Euc1 CC domain is required for dimerization. Y2H assay to test the requirement for the Euc1 CC domain for dimerization. Euc1-CCΔ: Δaa 104–129. Serial dilutions were spotted and cells were grown at 30°C for 2 or 6 days as indicated.

Appendix Figure S6.

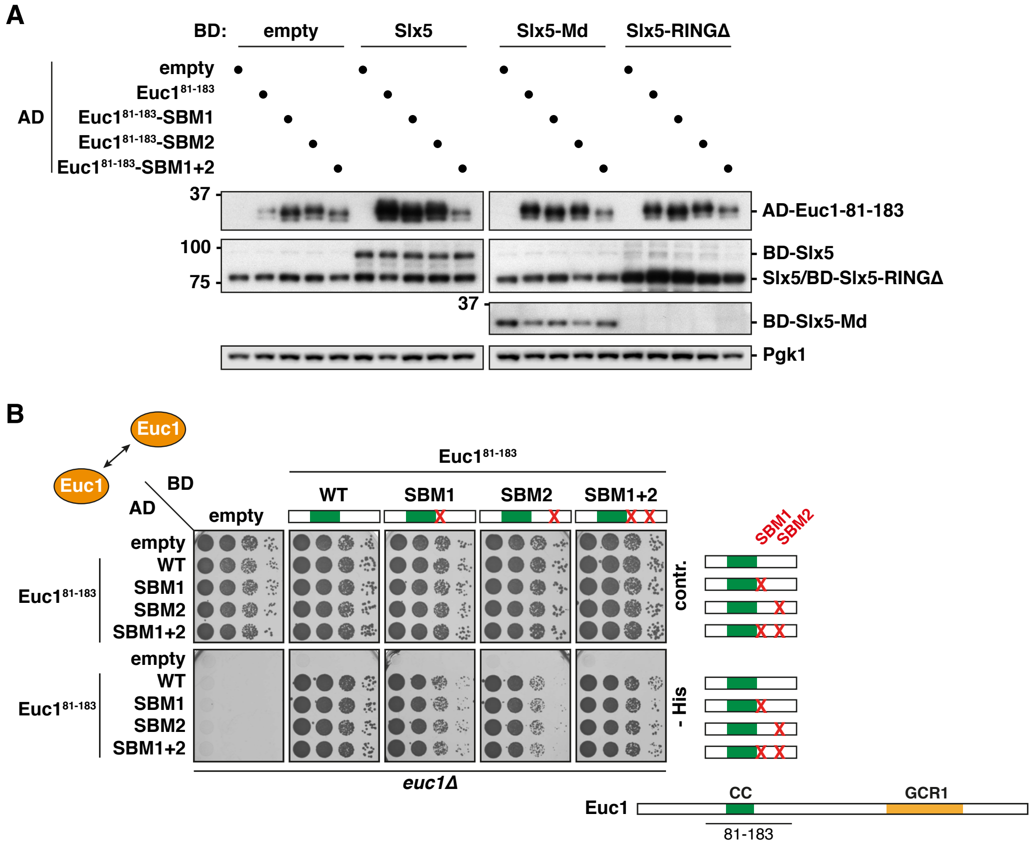

Appendix Fig. S6, related to Fig. 5

(A) Expression levels of the constructs expressed for Y2H assays in Fig. 5D and G analyzed by WB. AD-Euc1 constructs were probed with a Gal4-AD-specific antibody, BD-Slx5 constructs were probed with a polyclonal Slx5 antibody (raised against Slx5 aa 1–487). Note that for BD-Slx5-Md (aa 201–335) constructs levels cannot be directly compared with full length or Slx5-RINGΔ constructs, because the polyclonal Slx5 antibody might recognize more epitopes in the latter constructs. Pgk1 was probed to ensure equal amounts of cell material.

(B) Euc1-SBM1/2 constructs are proficient for dimerization. Y2H assay to test the effects of Slx5 binding site mutations on Euc1 dimerization.

Appendix Figure S7.

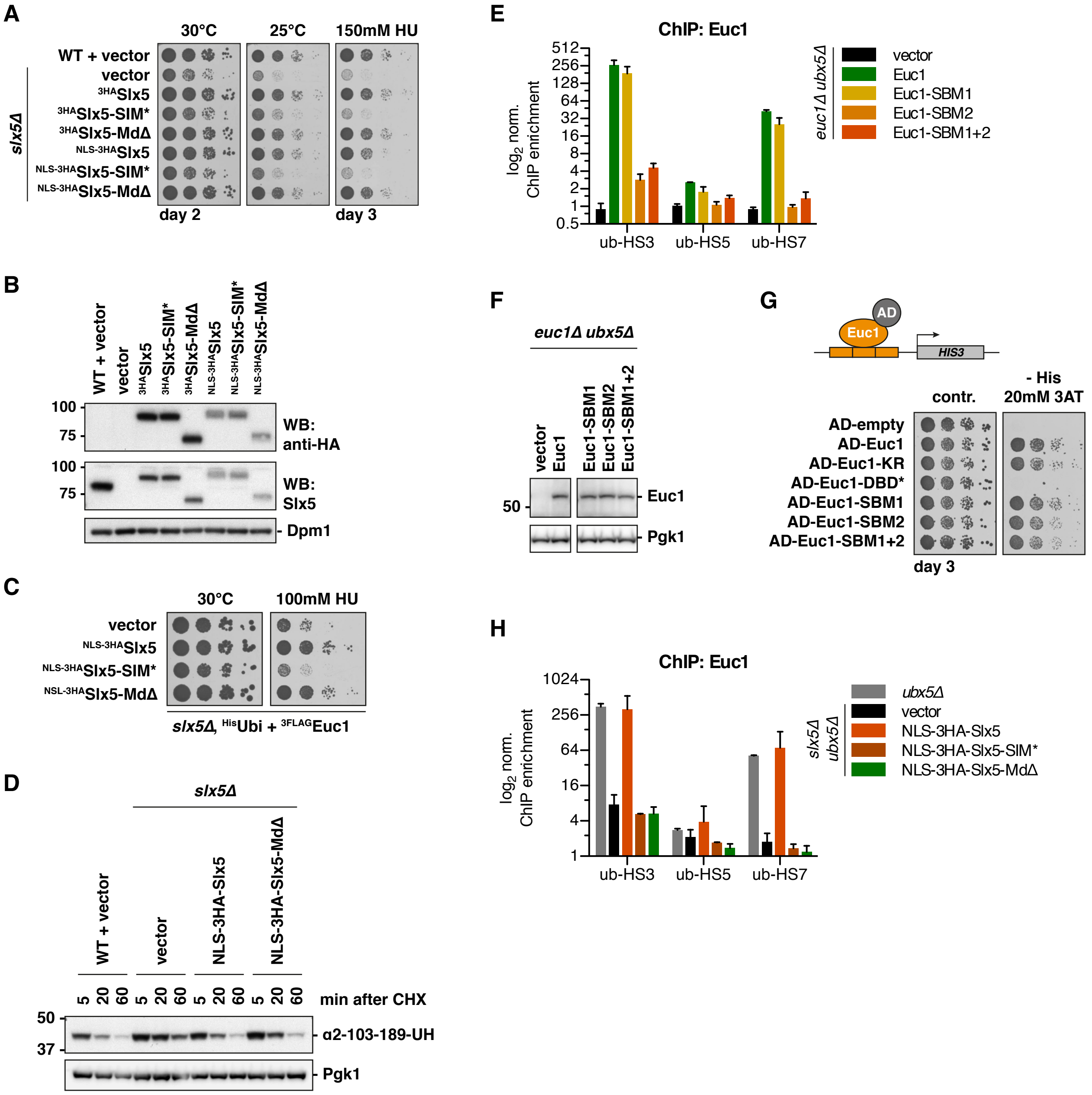

Appendix Fig. S7, related to Fig. 6

(A) The Slx5-Md is not required to rescue the *slx5Δ* replication stress phenotype or cold sensitivity. WT or *slx5Δ* cells were transformed with plasmids expressing the indicated constructs and spotted in serial dilutions. Cells were grown for 2 days (30°C, 25°C) or 3 days (150 mM hydroxyurea (HU) at 30°C). Note that addition of an N-terminal NLS did not affect the complementation.

(B) Expression levels of Slx5 constructs in cells used in (A) probed by WB against the HA-tag (top), Slx5 (middle) and Dpm1 as loading control (bottom).

(C) HU-complementation assay as described in (A) for cells used in Fig. 6B.

(D) The Slx5-Md is dispensable for Matα2 degradation. Degradation of a Matα2-fragment (α2-103-189-UH (Ura3-3HA)) that has previously been shown to rely on Slx5 for rapid degradation (Hickey & Hochstrasser, 2015) has been monitored in CHX-chase experiments (0.5 mg/ml cycloheximide from t=0). An *slx5Δ* strain was complemented with the indicated constructs expressed from plasmids under the *SLX5* promoter. WB for α2-103-189-UH was probed with an HA antibody and Pgk1 levels are shown as loading control.

(E) Euc1 Slx5-binding mutants (SBM1/SBM2) show reduced binding to ub-HSs. ChIP-qPCR analysis for Euc1 constructs. Data represent means ± SD ($n = 3$). See also (F) for expression levels and (G) for Y1H DNA-binding assay of Euc1-SBM1/2 constructs.

(F) Expression levels of Euc1 constructs in cells used in (E) and Fig. 6C-D probed by WB against Euc1. Pgk1 served as loading control. Sections were cropped from the same exposure.

(G) Euc1-SBM1/2 constructs bind the ub-HS-motif in Y1H assays. Gal4-AD fusions with the indicated constructs were used in a Y1H assay as described in Fig. 2E.

(H) Euc1 recruitment to ub-HS sites is impaired when Slx5-SIMs are mutated or the Slx5-Md is deleted. ChIP-qPCR analysis for Euc1 in the same cells as used for Fig. 6E-F. Data represent means ± SD ($n = 2$).

Appendix Figure S8.

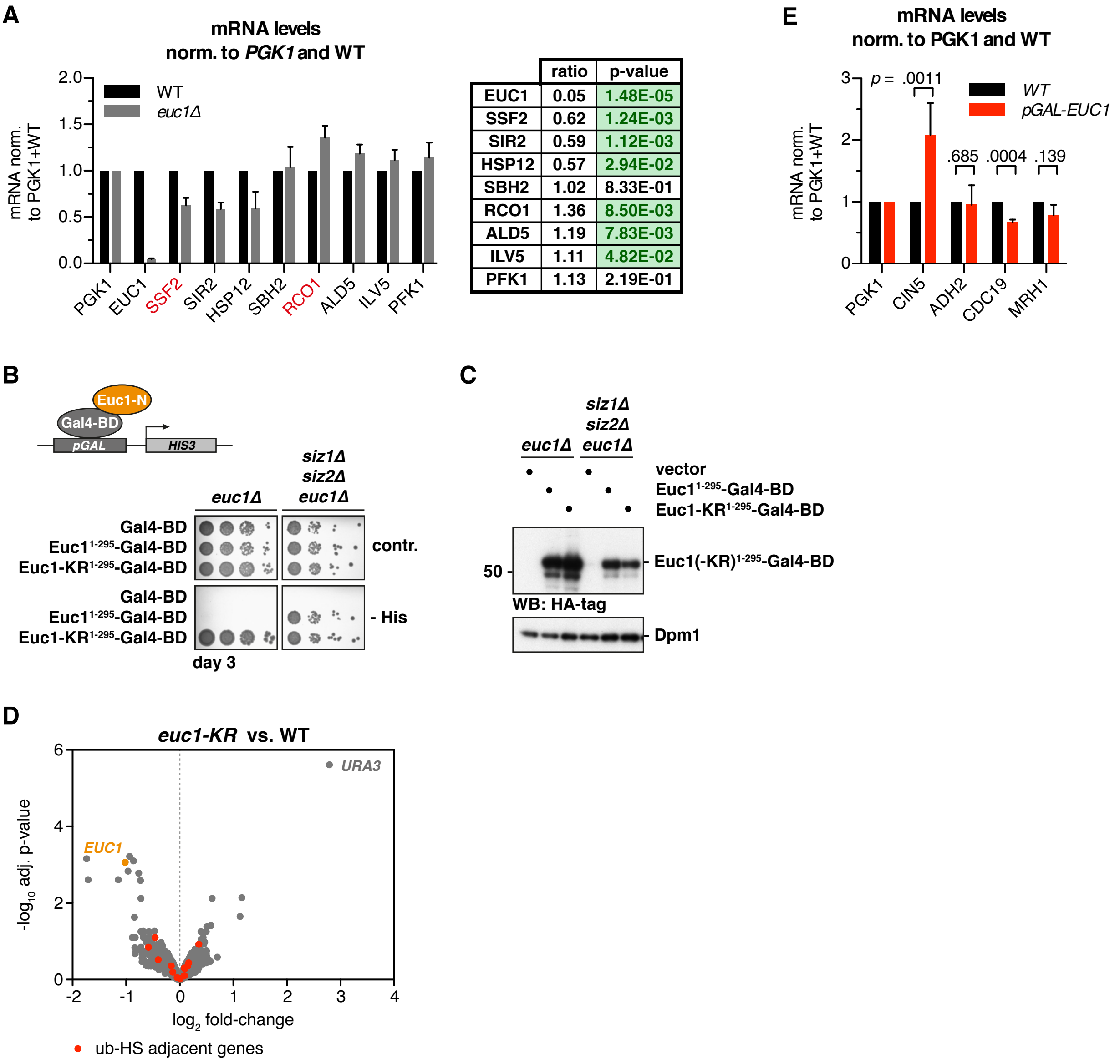

	ratio	p-value
EUC1	0.05	1.48E-05
SSF2	0.62	1.24E-03
SIR2	0.59	1.12E-03
HSP12	0.57	2.94E-02
SBH2	1.02	8.33E-01
RCO1	1.36	8.50E-03
ALD5	1.19	7.83E-03
ILV5	1.11	4.82E-02
PFK1	1.13	2.19E-01

Appendix Fig. S8, related to Fig. 7

(A) RT-qPCR quantification of genes deregulated in the *euc1Δ* transcriptome (Fig. 7A). Cells were grown in standard conditions and quantification was performed as for Fig. 3D, but normalization was performed with *PGK1*, because *ACT1* was mildly upregulated in *euc1Δ* cells in RNAseq. Quantification was performed on the same three replicate samples as used for RNAseq. Data represent means ± SD ($n = 3$), for statistical analysis an unpaired, two-tailed Student's *t*-test was performed.

(B) SUMOylation controls TF activity of an Euc1$^{1\text{-}295}$-Gal4-BD hybrid construct. Euc1-Gal4-BD fusion constructs as described in Appendix Fig. S4D were expressed and *HIS3*-activation was monitored. Data for Gal4-BD are reproduced from Appendix Fig. S4D for comparison.

(C) Expression levels of constructs used in (B) probed by an HA-antibody that recognizes an internal HA-tag upstream of Gal4-BD. Dpm1 served as loading control.

(D) Genes around the ub-HS sites are not differentially expressed in *euc1-KR* cells relative to WT cells. Volcano plots of a microarray-based transcriptome analysis of a *euc-KR* strain versus WT. Genes directly adjacent to the ub-HSs are depicted in red, irrespective of gene orientation and distance to the respective ub-HS. Triplicate experiments were analyzed on GeneChIP Yeast Genome 2.0 arrays (Affymetrix). Genes around the ub-HS sites are marked in red. *URA3* appears upregulated as it was used as a marker in the *euc1-KR* strain.

(E) RT-qPCR quantification as in (A) of genes misregulated in the *pGAL-EUC1* transcriptome (Fig. 7B). Data was obtained from an experiment independent of Fig. 7B. Data represent means ± SD ($n = 3$), for statistical analysis an unpaired, two-tailed Student's *t*-test was performed.

Appendix Figure S9.

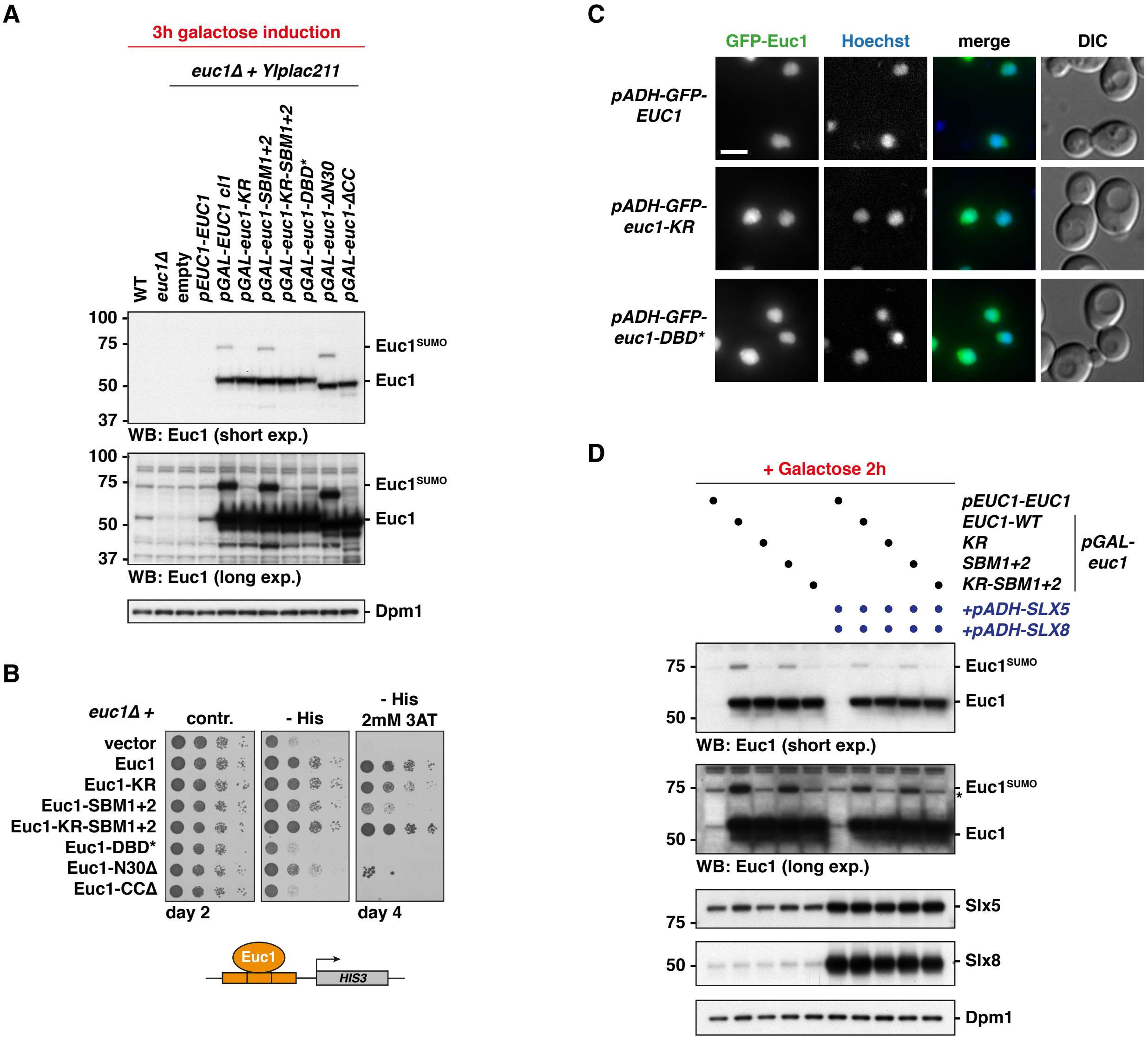

Appendix Fig. S9, related to Fig. 7

(A) WB analysis of galactose-induced (3h) *euc1*-alleles used in Fig. 7D.

(B) Reporter-gene assay as in Fig. 3C with *euc1*-alleles used in Fig. 7D, but expressed from plasmids and under the control of the endogenous *EUC1* promoter.

(C) Microscopy of *pADH-GFP-EUC1* (or *euc1-KR*, *euc1-DBD**) showing similar nuclear localization. Cells were counterstained with Hoechst 33342 dye to visualize DNA and maximum Z-projections of images recorded on a GE Deltavision Elite are shown. Scale bar 5μm.

(D) WB for strains used in Fig. 7F and EV4E after 2h galactose induction.

Appendix Figure S10.

A

Group	Strain	30°C	22°C	37°C	DMSO
	WT	4	4	4	4
	euc1Δ	4	4	4	4
H2A.Z/ SWR1-C chromatin remodeling	htz1Δ	4	3	2	2
	euc1Δ htz1Δ	4	2	1	1
	swr1Δ	4	3	3	2
	euc1Δ swr1Δ	4	3	2	1
	yaf9Δ	4	3	0	0
	euc1Δ yaf9Δ	4	2	0	0
Rpd3-HDAC core	rpd3Δ	3	3	0	1
	euc1Δ rpd3Δ	3	3	0	1
	sin3Δ	3	3	1	2
	euc1Δ sin3Δ	3	3	1	2
Rpd3L specific subunits	ume6Δ	3	2	1	2
	euc1Δ ume6Δ	3	2	1	2
	dep1Δ	4	4	3	3
	euc1Δ dep1Δ	4	4	1	2
	rxt2Δ	4	4	3	3
	euc1Δ rxt2Δ	4	4	2	2
	sds3Δ	4	4	2	2
	euc1Δ sds3Δ	4	4	0	1
Rpd3S specific subunits	rco1Δ	4	4	4	4
	euc1Δ rco1Δ	4	4	4	4
	eaf3Δ	4	4	4	4
	euc1Δ eaf3Δ	4	4	4	4
ubiquitin proteasome system	cim3-1	2	3	0	3
	euc1Δ cim3-1	2	3	0	3
	rpn12-1	2	2	0	1
	euc1Δ rpn12-1	1.5	1.5	0	1
	doa1Δ	4	4	4	4
	euc1Δ doa1Δ	4	4	4	4
	sem1Δ	4	4	0	3
	euc1Δ sem1Δ	4	4	0	3
	san1Δ	4	4	4	4
	euc1Δ san1Δ	4	4	4	4
mRNA processing/ splicing	npl3Δ	3	2	0	3
	euc1Δ npl3Δ	3	1	0	2
	snu66	4	3	4	4
	euc1Δ snu66Δ	4	3	4	4
	hub1Δ	4	4	4	4
	euc1Δ hub1Δ	4	4	4	4
	snt309Δ	1	1	0	1
	euc1Δ snt309Δ	1	1	0	1
H2B-Ub pathway	bre1Δ	4	3	1	1
	euc1Δ bre1Δ	4	3	1	1
	ubp8Δ	4	3	4	2
	euc1Δ ubp8Δ	4	3	4	2
	h2b-K123R	4	3	1	1
	euc1Δ h2b-K123R	4	3	1	1
	rad6Δ	3	3	1	1
	euc1Δ rad6Δ	3	3	1	0.5
others	stb5Δ	4	3	3	3
	euc1Δ stb5Δ	4	2	3	2
	mot3	4	3	4	4
	euc1Δ mot3Δ	4	3	4	4
	yef1Δ	4	4	4	4
	euc1Δ yef1Δ	4	4	4	4
	rpl6bΔ	3	3	4	3
	euc1Δ rpl6bΔ	3	3	4	3
	mco14Δ	4	4	4	4
	euc1Δ mco14Δ	4	4	4	4
	nup133Δ	3	3	0	2
	euc1Δ nup133Δ	3	3	0	2

growth relative to WT				
4	3	2	1	0
full				lethal

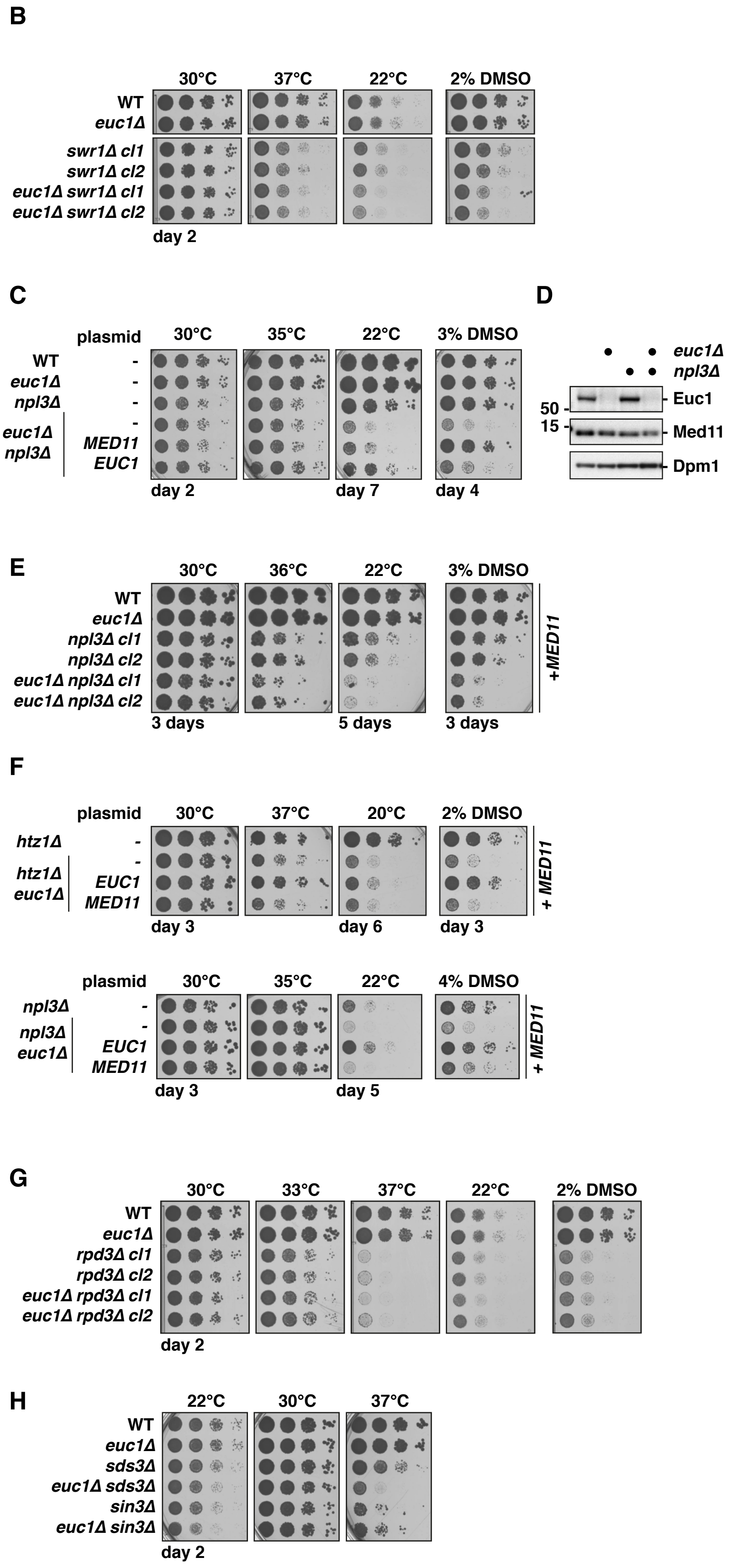

Appendix Fig. S10, related to Fig. 8

(A) Table summarizing genetic interactions manually tested in growth-based spotting assays as shown in Fig. 8A. Scores were given relative to WT growth in the respective conditions. For genetic interactions with *EUC1* (highlighted in red), at least two independent clones were tested. Note that all strains were generated from diploid strains via tetrad dissection and contained an extra copy of *MED11* (see C–F), except for *rpn12-1* strains.

(B) *EUC1* shows genetic interactions with *SWR1*. Spotting assay as described for Fig. 8A.

(C) Certain *euc1Δ* phenotypes can partially be rescued by introducing an extra copy of *MED11*. Plasmid complementation with *EUC1* or *MED11* with their endogenous promoters as indicated. *MED11* encodes for a subunit of the RNA Pol II mediator component, which is encoded by the ORF adjacent to *EUC1*. We noticed a mild downregulation of *MED11* in our transcriptome data and on protein level ((D), Fig. 7A). Therefore, we introduced an extra copy of *MED11* at the *URA3* locus (YIplac211) for our genetic analysis. See also (D-F).

(D) Med11 protein levels are mildly reduced in *euc1Δ npl3Δ* cells. WB against Euc1, Med11 and Dpm1.

(E) *EUC1* and *NPL3* show genetic interactions also in the presence of an extra copy of *MED11*. Spotting assay as described in Fig. 8A.

(F) Plasmid-borne *EUC1* expression rescues *euc1Δ* phenotypes. Indicated strains were transformed with *EUC1* or *MED11*-plasmids and grown on selective media.

(G-H) *EUC1* does not show genetic interactions with the Rpd3S/L core subunits *RPD3* and *SIN3*. Spotting assays as described in Fig. 8A. Note the stronger growth phenotypes of *rpd3Δ* and *sin3Δ* at elevated temperatures or on DMSO compared to *sds3Δ*.

Appendix Fig. S11, related to Fig. 8-9

(A) Genes deregulated in *euc1Δ* (top, see Fig. 7A) or upon *EUC1* overexpression (bottom, *pGAL-EUC1*, see Fig. 7B) show correlations with genes deregulated in published *rpd3Δ* and *set2Δ* transcriptomes. Genes up- or downregulated in the query datasets were marked in the gene rank plots of *euc1Δ* or *pGAL-EUC1* datasets. NES: normalized enrichment score (negative values for a correlation with *euc1Δ/pGAL-EUC1* downregulated genes, positive values for correlation with upregulated genes), padj: adjusted *p*-value. See Appendix Supplementary Methods for details.

(B) Euc1 is recruited to ub-hotspots upon heat stress. Statistical analysis of ChIP-qPCR data presented in Fig. 9A. For statistical analysis an unpaired, two-tailed Student's *t*-test was performed (n = 4).

(C) Euc1 is recruited to ub-hotspots in *htz1Δ* cells. ChIP-qPCR analysis of ubiquitin (K48), Euc1 and H2A.Z enrichments at selected ub-hotspots. *p*-values for comparisons of ubiquitin and Euc1 enrichments in WT ant *htz1Δ* are indicated. For ub-K48 and Euc1 experiments n = 3, for H2A.Z n = 2. For statistical analysis an unpaired, two-tailed Student's *t*-test was performed.

(D-E) Euc1 is recruited to ub-hotspots in *npl3Δ* cells upon cold stress. ChIP-qPCR for cells grown at 30°C, shifted to 25°C for 1h, or grown at 25°C. Note that ubiquitin is enriched at 25°C also at the Euc1-independent *ub-only-site1*, arguing for an Euc1-independent effect. Statistical analysis was performed for triplicate experiments (duplicates for *ub-only-site1*) as described in (B).

Appendix Figure S11.

A

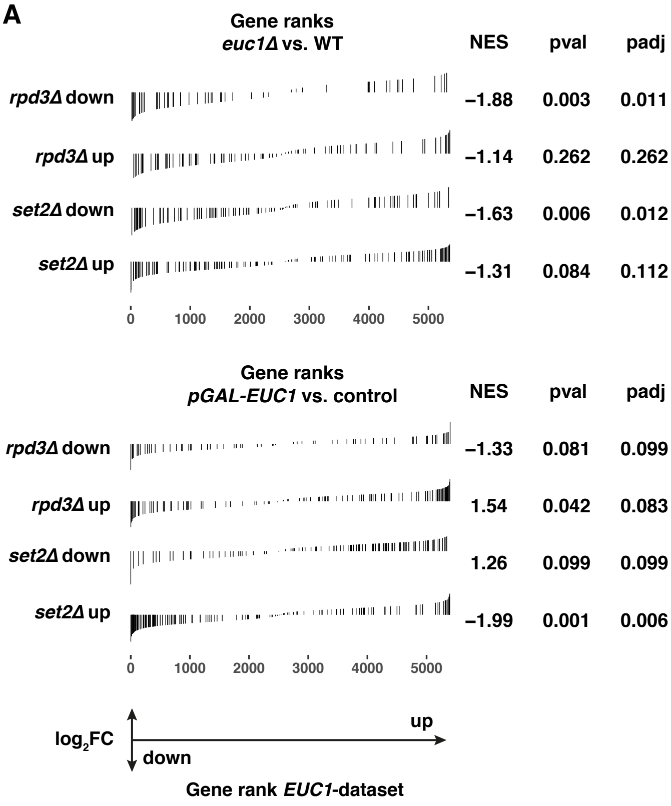

D

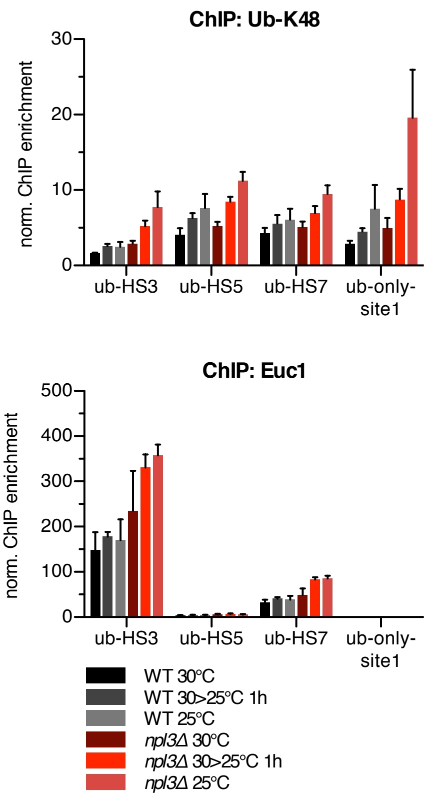

B

	30°C vs 37°C		WT vs. *sds3Δ*	
	WT	*sds3Δ*	30°C	37°C
	p-value ChIP ubiquitin (K48)			
ub-HS3	3.33E-01	5.81E-01	3.75E-01	7.50E-01
ub-HS5	1.97E-03	3.37E-03	1.40E-01	1.05E-01
ub-HS7	8.53E-04	2.76E-03	9.12E-01	5.45E-02
ub-only-site1	3.65E-03	7.12E-03	3.63E-01	2.61E-01
	p-value ChIP Euc1			
ub-HS3	5.90E-02	3.03E-03	5.07E-02	9.07E-03
ub-HS5	8.41E-02	2.83E-05	3.40E-01	1.56E-02
ub-HS7	2.40E-02	7.99E-05	7.56E-02	3.53E-02
ub-only-site1	2.15E-01	5.71E-01	4.19E-01	3.32E-02

E

	30°C vs 25°C		WT vs. *npl3Δ*	
	WT	*npl3Δ*	30°C	25°C
	p-value ChIP ubiquitin (K48)			
ub-HS3	6.94E-02	1.67E-02	4.94E-03	1.38E-02
ub-HS5	4.02E-02	1.34E-03	1.26E-01	4.71E-02
ub-HS7	1.27E-01	5.05E-03	2.23E-01	3.45E-02
ub-only-site1	1.71E-01	8.62E-02	1.65E-01	1.38E-01
	p-value ChIP Euc1			
ub-HS3	5.77E-01	8.21E-02	1.95E-01	3.48E-03
ub-HS5	5.47E-01	8.20E-01	1.10E-01	1.70E-01
ub-HS7	3.78E-01	1.73E-02	1.29E-01	1.84E-03
ub-only-site1	4.66E-01	8.55E-02	9.83E-01	2.36E-01

C

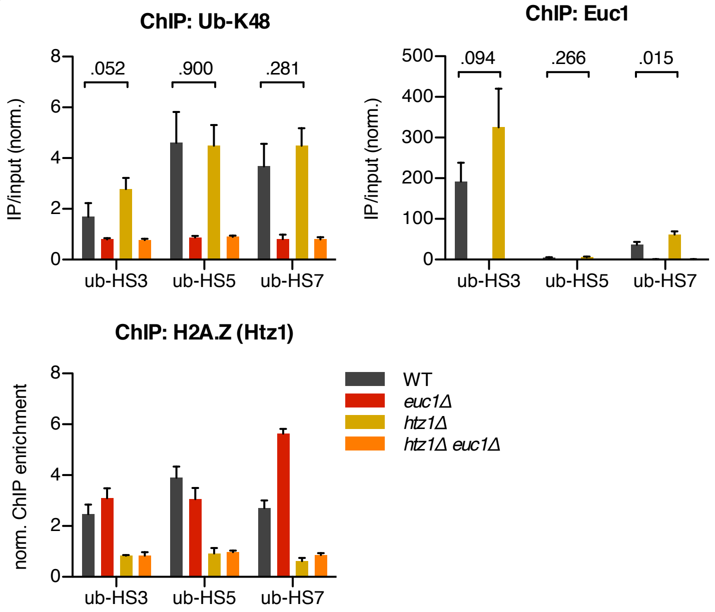

Appendix Figure S12.

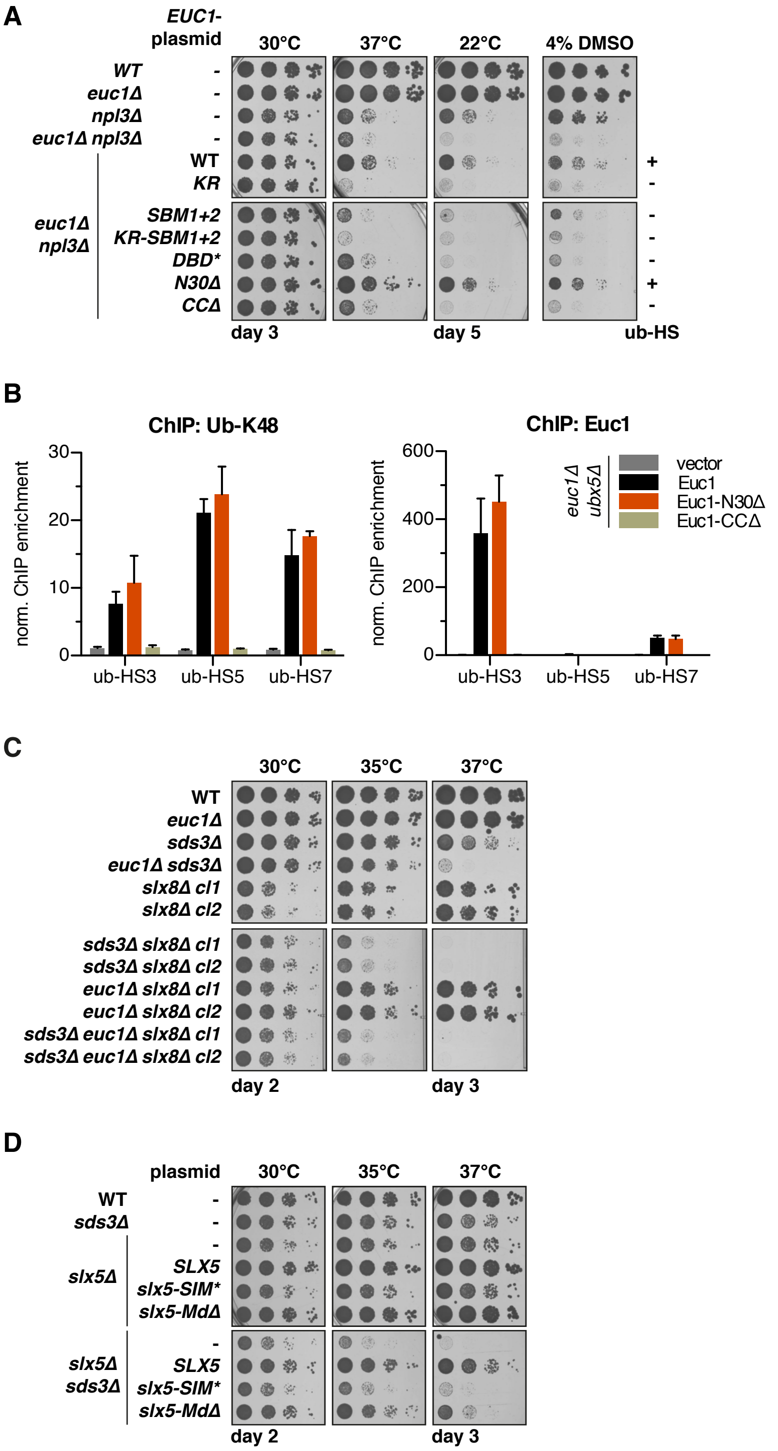

Appendix Fig. S12, related to Fig. 9

(A) Complementation of *euc1Δ npl3Δ* heat, cold, and DMSO sensitivity phenotypes with plasmid-borne *EUC1* alleles as in Fig. 9B.

(B) Euc1-N30Δ, but not Euc1-CCΔ is proficient in forming ub-hotspots. ChIP-qPCR quantification of ubiquitin and Euc1 for *euc1Δ ubx5Δ* cells complemented with the indicated plasmids. Data represent means ± SD ($n = 3$), for Euc1-CCΔ $n = 2$.

(C) *SLX8* shows genetic interactions with *SDS3* upon heat stress. Spotting as in Fig. 9C, but with *slx8Δ* strains as indicated.

(D) The Slx5-SIMs and Slx5-Md are required for full complementation of *slx5Δ sds3Δ* phenotypes. Strains were transformed with plasmids expressing *SLX5* alleles under the endogenous promoter and spotted on selective media.

Appendix Supplementary Methods

Yeast methods and molecular biology

All yeast strains are isogenic to DF5 (Finley *et al*, 1987), unless stated otherwise, and are listed in Appendix Table S1. Genetic modifications were performed as described (Knop *et al*, 1999; Janke *et al*, 2004) and cells were grown at 30°C if not indicated otherwise. Cells were grown in YPD medium (2% glucose) or synthetic complete medium lacking individual amino acids to maintain selection for transformed plasmids. For galactose induction, cells were cultured in YP + 2% raffinose (or 3% lactic acid for ChIP-chip experiments) and galactose was added to a final concentration of 2%. Whole cell protein extracts were prepared by alkaline lysis followed by TCA precipitation as described (Knop *et al*, 1999) and protein levels were analyzed by Western blotting. For quantification of band intensities, a LI-COR Odyssey Fc instrument was used to detect a chemiluminescence signal (HRP/ECL) and the ImageStudio Lite software (LI-COR) for image analysis. For quantification in Fig. 6B, film was scanned and quantified. For cycloheximide (CHX) chase experiments, mid-log phase cells were pelleted gently, resuspended in fresh medium supplemented with 0.5mg/ml CHX and samples were taken at the indicated time-points.

For all temperature sensitive alleles, cells were grown at the semi-permissive temperature of 30°C unless stated otherwise, since no further increase of ubiquitin enrichment at ub-hotspots was observed at higher temperatures. For mutations of the essential *CDC48*-gene, we initially used the *cdc48-6* allele, however, we noticed that this strain harbors a partial duplication of chromosome XIII, which includes the *EUC1* gene. Therefore, the *cdc48-3* allele was introduced into DF5 with a *LEU2* marker cassette downstream.

Cloning methods used standard protocols or the Gibson Assembly Master Mix (NEB).

Recombinant proteins and antibodies

6His-Slx5 was purified as described (Yang *et al*, 2006), GST and GST-Euc1 were expressed from pGEX4T3 vectors (GE Healthcare) and purified by NiNTA-agarose (Qiagen) or Glutathion-sepharose (GE Healthcare).

Antibodies against the specified proteins for Western blotting were used as follows: polyclonal rabbit antibodies were raised against Euc1 (aa 292-462), Slx5 (aa 1-487) and Smt3 (Hoege *et al*, 2002); commercial antibodies were against ubiquitin (P4D1, Cell Signaling Technology), Dpm1 (A6429) and Pgk1 (#459250) form Invitrogen/Life Technologies, FLAG-HRP (M2, Sigma), H2B-ub (D11, Cell Signaling Technology), Gal4-AD (ab135398,

abcam), HA-tag (3F10, Roche), Med11 (ab221200, abcam). For antibodies used in ChIP, see below.

GST-pulldown assay

For GST-pulldown assays, indicated proteins were mixed in binding buffer (50mM Tris pH 7.5, 5mM DTT, 150mM NaCl, 0.5% NP-40, 20mM imidazole) and incubated 30 min at room temperature. Input samples were set aside and magnetic glutathione beads (ThermoFisher) were added and samples rotated for one hour. Beads were washed three times with wash buffer (as binding buffer, but 300mM NaCl) and eluted with 5mM glutathione. Finally, samples were separated by SDS-PAGE and stained with PageBlue (ThermoFisher).

Chromatin immunoprecipitation (ChIP)

ChIP was performed based on a protocol (Aparicio *et al*, 2005), with minor modifications as described (Kalocsay *et al*, 2009). Briefly, for each IP yeast cells equivalent to 75 OD_{600}-units were crosslinked with formaldehyde (1% final) for 16 minutes and the reaction was quenched by adding glycine (325mM final). Cells were lysed by beat-beating (MM301, Retsch GmbH) with zirconia/silica beads (BioSpec Inc.), chromatin was isolated and sheared to 200-500 bp fragments using a Bioruptor UCD-200 sonication system (Diagenode). Cell debris was pelleted by centrifugation, input samples were collected and chromatin was subjected to immunoprecipitation with Protein A Sepharose CL-4B (GE Healthcare) and specific antibodies as follows: ubiquitin (clone FK2, Merck/Millipore), ubiquitin-K48-specific (Apu2, Merck/Millipore), Myc-tag (9E10, Sigma), HA-tag (ab9110, abcam), unspecific rabbit IgG (Bethyl Laboratories Inc.) and specific rabbit antibodies raised against Euc1 (aa 292-462) or Slx5 (aa 1-487). Enriched DNA was de-crosslinked and subjected to Proteinase K digest (Sigma), purified (Qiagen PCR purification kit) and subjected to qPCR-analysis (KAPA SYBR FAST, Roche) on a Light Cycler 480 instrument (Roche) or processed for genome-wide quantification using NimbleGen arrays as detailed below.

For quantification by qPCR, triplicate measurements were performed and ChIP-DNA was normalized to input DNA for each tested locus and then normalized to a control region on chromosome II (*TOS1* promoter) that showed low fluctuations in ChIP-chip experiments for ubiquitin, Slx8 and Euc1. Hence, background levels were defined as 1. Primers used for qPCR are listed in Appendix Table S3.

ChIP-chip

ChIP-chip was performed as described before (Kalocsay *et al*, 2009; Renkawitz *et al*, 2013). Briefly, input and ChIP DNA samples were treated with RNase (Sigma) and amplified with the GenomePlex Complete Whole Genome Amplification kit (Sigma) in two steps as described (O'Geen *et al*, 2006). Labeling of input and ChIP DNA (Cy3 or Cy5), hybridization to *S. cerevisiae* tiling arrays, array scanning and raw data extraction was performed by Source BioScience Berlin (formerly imaGenes, NimbleGen ChIP-chip service). Custom-designed c12plex NimbleGen arrays with 84 bp median genomic probe spacing were used and only unique probes were analysed. A dye-swap was included for replicate experiments and genome-wide binding profiles were generated from two independent experiments (except for ub-FK2 *rad6*Δ and IgG WT profiles in Fig. 1A, performed once). Data presented in figures is on log_2-scale, normalized to input DNA and plots were generated using IGB (Integrated Genome Browser).

Bioinformatic analysis of ChIP-chip data

Data analysis was performed using R/Bioconductor. Raw tiling array signals were log_2 transformed and quantile normalized (library 'preprocessCore'). For peak calling we first computed a t statistic using the 90% rule of Efron (function 'efron.stat', library 'st') on IP versus background for the signals of each probe. We then determined the local false discovery rate (lfdr, library 'locfdr') on the t values for each probe. In case of $n = 1$ sample we instead calculated the local false discovery rate on the IP over background ratio for each probe. Probes less than 350 bp apart with a lfdr < 0.2 and a log_2 (IP/background ratio) > 0.5 (1 in case of Euc1/Ymr111c) were merged. Merged regions broader than 500 bp were defined as peaks (enriched regions, ER).

Ub-hotspot DNA-motif prediction

1-2 kb regions around the ub-hotspots were used as input for DNA-motif prediction using the MEME online tool (Bailey *et al*, 2009). The following sequences were used as input: ub-HS1 (left) Chr III 123330-124330, ub-HS1 (right) Chr III 125000-126000, ub-HS2 Chr IV 358000-359000, ub-HS3 Chr IV 1086700-1087700, ub-HS4 Chr XIII 308500-310500, ub-HS5 Chr XIII 413400-414400, ub-HS6 Chr XIII 433200-434200, ub-HS7 Chr XV 167800-168800.

mRNA-quantification and transcriptome analysis

Yeast total RNA was isolated form 1.5-4 OD_{600} units of yeast cells using the RNeasy Kit (Qiagen) following the manufacturer's instruction, with beat-beating for cell lysis and DNase I digest performed on-column. RNAseq based transcriptome analysis is detailed in the main Materials and Methods section, microarray based transcriptome analysis was performed in biological triplicates using GeneChIP Yeast Genome 2.0 arrays (Affymetrix) according to the manufacturer's instructions. Samples were processed using the GeneChip 3' IVT Express Kit and GeneChip Hybridization, Wash, and Stain Kit (both Affymetrix) and arrays were scanned on a GeneChip scanner 3000 (Affymetrix). For data analysis, R/Bioconductor was used (Gentleman *et al*, 2004). First, raw gene expression intensities were normalized by Robust Multiarray Analysis (RMA) (Irizarry *et al*, 2003) and the affyPLM and simpleaffy packages were used for quality control (Gentleman *et al*, 2004; Wilson & Miller, 2005). Limma and affy microarray packages (Gautier *et al*, 2004) were then used for low-level analysis and gene annotation was obtained from R/Bioconductor metadata packages and biomaRt repository (Durinck *et al*, 2005; 2009). For differential expression comparison between two strains a fixed linear model was fitted for individual features to estimate expression differences between the two sample groups. Standard errors of M-values were moderated by using an empirical Bayes approach. Finally, a moderated t-statistics, raw and adjusted *p*-values (Benjamini and Hochberg FDR control) were obtained for each feature. A 2-fold change was used as cut-off for biological significance.

For qPCR-based quantification of mRNA, isolated RNA was treated with an additional in solution DNase I digest (Qiagen) and precipitated with NaOAc pH 5.2 (0.3M final) and 70% EtOH (final) or DNase I was inactivated by denaturation at 75°C, 10 min. A Transcriptor First Strand cDNA Synthesis Kit (Roche) was used according to the manufacturer's instructions for cDNA synthesis and qPCR was performed on a Light Cycler 480 instrument (Roche). *HIS3* mRNA-levels were normalized to *ACT1* or *PGK1*-levels for each sample and then normalized to the reference sample.

For gene enrichment analysis, we defined sets of up- and downregulated genes upon deletion of *RPD3* (GEO: GSE67151) and *SET2* (GEO: GSE89265) by an adjusted *p*-value cutoff of < 0.1 and a $\log_2$ fold-change cutoff of 1.5 (*rpd3*Δ) and 2 (*set2*Δ). The four gene sets were tested for enrichment on the expression changes observed upon *EUC1* deletion and overexpression (*pGAL-EUC1*) using fgsea (v. 1.8.0) with 1000 permutations.

Slx5 binding site prediction

To identify the putative sites responsible for interaction with Slx5, we ran a *de novo* motif prediction with a development version of HH-MOTiF (Prytuliak *et al*, 2017). The input dataset consisted of putative Slx5 substrates identified in a mass spectrometry experiment. Additionally, Ymr111c/Euc1 as putative Slx5/Slx8 substrate was included. From the obtained candidate sites, only those containing an instance in the region amino acid 81-183 of Ymr111c/Euc1 were retained. As a result, only three motif candidates passed the filtering procedure. Upon replacement of several charged/hydrophobic residues with alanine in Euc1 aa 81-183, two binding site candidates showed decreased interaction with Slx5 in Y2H experiments (Slx5-binding mutant 1/2, SBM1/2).

Yeast two-hybrid (Y2H) assay

In vivo protein interactions were tested with the yeast two-hybrid system as described (James *et al*, 1996). Briefly, plasmids encoding Gal4 activation domain (AD) and binding domain (BD) fusion proteins were transformed in Y2H reporter strains (PJ69-7a or isogenic strains with indicated deletions (James *et al*, 1996)), several clones were mixed, adjusted to OD_{600}=0.5 and spotted on control or selective media (lacking histidine) and incubated at 30°C for 2-5 days as indicated. Optionally, 5-fold serial dilutions were spotted.

Co-immunoprecipitation

Two alternative methods were used for immunoprecipitation (IP) of Euc1. For Fig. 4B, native cell lysates from 200 OD_{600} units of mid-log phase yeast cells were prepared in IP lysis buffer (50mM Tris pH 7.5, 150mM NaCl, 10% glycerol, 2mM $MgCl_2$, 0.5% NP-40, 1mg/ml Pefabloc SC (Roche), 1x complete EDTA-free protease inhibitor cocktail (Roche), 20mM N-ethylmaleimide (NEM)). Cells were lysed by beat-beating (MM301, Retsch GmbH) with zirconia/silica beads (BioSpec Inc.), chromatin was sheared using a Bioruptor UCD-200 sonication system (Diagenode) and lysates were cleared by centrifugation (20000g, 8 min). Euc1 was captured with anti-Euc1 antibody and Protein A agarose (Roche) for 2 hours at 4°C, beads were washed with IP lysis buffer, proteins eluted and analyzed by WB.

For FLAG-IPs (Fig. 5C and E), 300 OD_{600} units of cells were resuspended in FLAG-IP buffer (100mM HEPES-KOH pH7.6, 200mM KOAc, 0.1% NP-40, 10% glycerol, 2mM β-mercaptoethanol, 2mM $MgCl_2$, 1mg/ml Pefabloc SC (Roche), 1x complete EDTA-free protease inhibitor cocktail (Roche), 20mM N-ethylmaleimide (NEM)), snap-frozen in liquid N_2 and broken up in a Freezer/Mill (FM6870, SPEX SamplePrep). DNA and RNA was

digested by incubation with 1.5µl benzonase (Merck/Millipore) and lysates were cleared by centrifugation (2000g, 20 min). For IP of ^{3FLAG}Euc1 (2 hours 4°C), anti-FLAG resin (M2, Sigma) was used, beads were washed with FLAG-IP buffer and eluted proteins were analyzed by WB.

Appendix Table S1. Yeast strains used in this study.

Diploid strains marked with (*) were sporulated and haploid strains were used for testing of genetic interactions. Diploid strains are available on request.

Strain	Relevant Genotype	Ref./Source
DF5	*trp1-1 ura3-52 his3Δ200 leu2-3,11 lys2-801*	(Finley *et al*, 1987)
MJK183	*DF5, MATa htb2::hphNT1; Htb1-K123R-tADH::kanMX4*	I. Psakhye
Y0066	*DF5, MATa rad6::HIS3*	(Hoege *et al*, 2002)
Y0649	*DF5, MATa cdc48-6*	(Rape *et al*, 2001)
Y0650	*DF5, MATα cdc48-6*	(Rape *et al*, 2001)
MJK503	*DF5, MATa cdc48-3::LEU2*	This study
Y2305	*DF5, MATa ump1::hphNT1*	Jentsch strain collection
Y1912	*DF5, MATa rad23::kanMX4 dsk2::kanMX4*	(Richly *et al*, 2005)
Y0472	*DF5, MATa ufd1-2*	(Rape *et al*, 2001)
Y0802	*DF5, MATa npl4-1*	(Rape *et al*, 2001)
YAB1729	*DF5, MATa shp1-7*	(Böhm & Buchberger, 2013)
MJK100	*DF5, MATa ubx2::kanMX4*	A. Buchberger
MJK101	*DF5, MATa ubx3::kanMX4*	A. Buchberger
Y0845	*DF5, MATa ubx4::hisMX6*	Jentsch strain collection
MJK102	*DF5, MATa ubx5::kanMX4*	A. Buchberger
MJK103	*DF5, MATa ubx6::kanMX4*	A. Buchberger
MJK104	*DF5, MATa ubx7::kanMX4*	A. Buchberger
Y3382	*DF5, MATα vms1::kanMX4*	Jentsch strain collection
Y0597	*DF5, MATa ufd2::LEU2*	(Koegl *et al*, 1999)
Y0578	*DF5, MATa ufd3::HIS3*	(Rumpf & Jentsch, 2006)
Y1908	*DF5, MATa otu1::kanMX4*	(Rumpf & Jentsch, 2006)
MJK253	*DF5, MATα cdc48-6 YIplac128-empty::LEU2*	This study
MJK256	*DF5, MATα cdc48-6 YIplac128-Ub-HS4::LEU2*	This study
MJK257	*DF5, MATα cdc48-6 YIplac128-Ub-HS4-F1::LEU2*	This study
MJK258	*DF5, MATα cdc48-6 YIplac128-Ub-HS4-F2::LEU2*	This study
MJK259	*DF5, MATα cdc48-6 YIplac128-Ub-HS4-F3::LEU2*	This study
MJK260	*DF5, MATα cdc48-6 YIplac128-Ub-HS4-F4::LEU2*	This study
MJK303	*DF5, MATα cdc48-6 YIplac128-Ub-HS4-F5::LEU2*	This study
MJK325	*DF5, MATα cdc48-6 YIplac128-Ub-HS4-F6::LEU2*	This study
MJK354	*DF5, MATα cdc48-6 YIplac128-Ub-HS4-F7::LEU2*	This study
MJK355	*DF5, MATα cdc48-6 YIplac128-Ub-HS4-F7-Mut::LEU2*	This study
MJK337	*DF5, MATα cdc48-6 YIplac128-Ub-HS4-F8::LEU2*	This study
YM4271	*MATa, ura3-52, his3-200, ade2-101, ade5, lys2-801, leu2-3, 112, trp1-901, tyr1-501, gal4D, gal8D, ade5::hisG*	Clontech
MJK391	*YM4271, ubx5::hphNT1 YIplac211-3xUb-HS4-F7-min.promoter-HIS3::URA3*	This study
MJK409	*YM4271, ubx5::hphNT1 YIplac211-3xUb-HS4-F7-Mut-min.promoter-HIS3::URA3*	This study
MJK447	*YM4271, ubx5::hphNT1 YIplac211-3xUb-HS4-F7-min.promoter-HIS3::URA3 euc1::natMT2*	This study
MJK407	*DF5, MATa ubx5::kanMX4 yfr006w::natNT2*	This study
MJK450	*DF5, MATa ubx5::kanMX4 euc1::natNT2*	This study
MJK448	*DF5, MATa euc1::natNT2*	This study
PJ69-7A	*trp-901-, leu2-3,112 ura3-53 his3-200 gal4 gal80 GAL1::HIS*	(James *et al*, 1996)

	GAL2-ADE2 met2::GAL7-lacZ	
MH093	*PJ69-7A, euc1::natNT2*	This study
MH443	*PJ69-7A, Slx8::caURA3MX4*	This study
MH719	*PJ69-7A, euc1::hphNT1*	This study
MH725	*PJ69-7A, siz1::kanMX4 siz2::natNT2 euc1::hphNT1*	This study
MJK531	*DF5, MATa siz1::HIS3*	I. Psakhye
MJK532	*DF5, MATa siz2::HIS3*	I. Psakhye
Y0439	*DF5, MATa ubc9-1::URA3*	Jentsch strain collection
Y3061	*DF5, MATa siz1::HIS3 siz2::HIS3*	Jentsch strain collection
MJK612	*DF5, MATa euc1-K231R::URA3*	This study
MJK460	*DF5, MATa siz1::HIS3 siz2::HIS3 ubx5::hphNT1*	This study
MJK611	*DF5, MATa ubc9-1::URA3 ubx5::natNT2*	This study
MJK616	*DF5, MATa euc1-K231R::URA3 ubx5::natNT2*	This study
MJK567	*DF5, MATa SLX8-9MYC::kanMX4*	This study
MJK569	*DF5, MATa SLX8-9MYC::kanMX4 euc1::natNT2*	This study
MJK590	*DF5, MATa SLX8-9MYC::kanMX4 siz1::HIS3 siz2::HIS3*	This study
MJK609	*DF5, MATa SLX8-9MYC::kanMX4 ubc9-1::URA3*	This study
MJK617	*DF5, MATa SLX8-9MYC::kanMX4 euc1-K231R::URA3*	This study
MJK571	*DF5, MATa Slx8-9myc::kanMX4 cdc48-3::LEU2*	This study
MJK579	*DF5, MATa slx5::HIS3MX6*	This study
MJK595	*DF5, MATa slx8::hphNT1*	This study
MJK604	*DF5, MATa pSMT3-smt3KRall-tSMT3*	I. Psakhye
MJK621	*DF5, MATa pSMT3-smt3KRall-tSMT3 ubx5::NatMX*	This study
MJK624	*DF5, MATa slx5::natNT2 ubx5::kanMX4*	This study
MH491	*DF5, MATa pADH-3HA-Slx5::NatNT2*	This study
MH499	*DF5, MATa pADH-3HA-Slx5::NatNT2 cdc48-3::LEU2*	This study
MH470	*DF5, MATa pADH-3HA-Slx5::NatNT2 euc1::kanMX4*	This study
MH497	*DF5, MATa pADH-3HA-Slx5::NatNT2 Euc1-K231R::URA3*	This study
MH493	*DF5, MATa pADH-3HA-Slx5::NatNT2 slx8::HIS3MX4*	This study
MH489	*DF5, MATa slx5::HIS3MX6 Slx8-9myc::kanMX4*	This study
MH745	*DF5, MATa euc1::kanMX4*	This study
MH743	*DF5, MATα euc1::kanMX4 pADH-HisSUMO-tADH::URA3*	This study
MH744	*DF5, MATa euc1::kanMX4 pADH-HisUb-tADH::URA3*	This study
MH747	*DF5, MATa euc1::kanMX4 Slx8-C206,209S::NatNT2 pADH-HisUb-tADH::URA3*	This study
MH649	*DF5, MATa euc1::kanMX4 slx8-C206S,C209S-tADH::NatNT2*	This study
MH845	*DF5, MATa euc1::kanMX4 pADH-HisUbi-tADH::URA3 slx5::HIS3MX4*	This study
MH850	*DF5, MATa euc1::kanMX4 pADH-HisUbi-tADH::URA3 cdc48-3::LEU2*	This study
MH629-1a1	*DF5, MATa EUC1-tADH::caURA3*	This study
MH630-2a1	*DF5, MATa euc1-K231R-tADH::caURA3*	This study
MH632-2a1	*DF5, MATa euc1-W333A,R334A-tADH::caURA3*	This study
MHY501	*MATα his3-Δ200 leu2-3,112 ura3-52 lys2-801 trp1-1*	(Chen *et al*, 1993)
MHY3712	*MATα his3-Δ200 leu2-3,112 ura3-52 lys2-801 trp1-1 slx5::kanMX4*	(Xie *et al*, 2007)
MH1047	*DF5, Mata YIplac211-MED11::URA3*	*This study*
MH1074	*DF5, Mata YIplac211-MED11::URA3 euc1::natNT2*	*This study*
MH1172	*DF5, Mata YIplac211-MED11::URA3 htz1::hphNT1*	*This study*
MH1173	*DF5, Mata YIplac211-MED11::URA3 htz1::hphNT1 euc1::natNT2*	*This study*
MH1174	*DF5, Mata YIplac211-MED11::URA3 stb5::hphNT1*	*This study*

MH1175	*DF5, Mata YIplac211-MED11::URA3 euc1::natNT2 stb5::hphNT1*	*This study*
MH1221	*DF5, Mata YIplac211-MED11::URA3 npl3::hphNT1*	*This study*
MH1222	*DF5, Mata YIplac211-MED11::URA3 euc1::natNT2 npl3::hphNT1*	*This study*
MH1158	*DF5, Mata YIplac211-MED11::URA3 dep1::kanMX4*	*This study*
MH1159	*DF5, Mata YIplac211-MED11::URA3 euc1::natNT2 dep1::kanMX4*	*This study*
MH1160	*DF5, Mata YIplac211-MED11::URA3 rxt2::kanMX4*	*This study*
MH1161	*DF5, Mata YIplac211-MED11::URA3 euc1::natNT2 rxt2::kanMX4*	*This study*
MH1164	*DF5, Mata YIplac211-MED11::URA3 sds3::kanMX4*	*This study*
MH1165	*DF5, Mata YIplac211-MED11::URA3 euc1::natNT2 sds3::kanMX4*	*This study*
MH1229	*DF5, Mata YIplac211-MED11::URA3 rco1::hph*	*This study*
MH1230	*DF5, Mata YIplac211-MED11::URA3 euc1::natNT2 rco1::hph*	*This study*
MH1231	*DF5, Mata YIplac211-MED11::URA3 sds3::kanMX4 rco1::hph*	*This study*
MH1232	*DF5, Mata YIplac211-MED11::URA3 euc1::natNT2 sds3::kanMX4 rco1::hph*	*This study*
MH1233	*DF5, Mata YIplac211-MED11::URA3 eaf3::hph*	*This study*
MH1234	*DF5, Mata YIplac211-MED11::URA3 euc1::natNT2 eaf3::hph*	*This study*
MH1235	*DF5, Mata YIplac211-MED11::URA3 sds3::kanMX4 eaf3::hph*	*This study*
MH1236	*DF5, Mata YIplac211-MED11::URA3 euc1::natNT2 sds3::kanMX4 eaf3::hph*	*This study*
MH1189	*DF5, Mata YIplac211-MED11::URA3 swr1::hphNT1*	*This study*
MH1190	*DF5, Mata YIplac211-MED11::URA3 euc1::natNT2 swr1::hphNT1*	*This study*
MH1237	*DF5, Mata YIplac211-MED11::URA3 yaf9::hph*	*This study*
MH1238	*DF5, Mata YIplac211-MED11::URA3 euc1::natNT2 yaf9::hph*	*This study*
MH578	*DF5, Mata npl3::hphNT1*	*This study*
MH580	*DF5, Mata npl3::hphNT1 euc1::natNT2*	*This study*
MH1162	*DF5, Mata YIplac211-MED11::URA3 sin3::kanMX4*	*This study*
MH1163	*DF5, Mata YIplac211-MED11::URA3 euc1::natNT2 sin3::kanMX4*	*This study*
MH1203	*DF5, Mata YIplac211-MED11::URA3 rpd3::kanMX4*	*This study*
MH1204	*DF5, Mata YIplac211-MED11::URA3 euc1::natNT2 rpd3::kanMX4*	*This study*
MH1151*	*DF5, Mata/α YIplac211-MED11::URA3 euc1::natNT2 ume6::kanMX4*	*This study*
MH1178*	*DF5, Mata/α cim3-1 YIplac211-MED11::URA3 euc1::natNT2*	*This study*
Y0351	*DF5, Matα ura3, his3, trp1, rpn12-1*	*Kominami 1994*
MH1184	*DF5, Matα ura3, his3, trp1, rpn12-1 euc1::natNT2*	*This study*
MH1180*	*DF5, Mata/α YIplac211-MED11::URA3 euc1::natNT2 doa1::kanMX4*	*This study*
MH1181*	*DF5, Mata/α YIplac211-MED11::URA3 euc1::natNT2 sem1::kanMX4*	*This study*
MH1182*	*DF5, Mata/α YIplac211-MED11::URA3 euc1::natNT2 san1::kanMX4*	*This study*
MH1126*	*DF5, Mata/α YIplac211-MED11::URA3 euc1::natNT2 snu66::kanMX6*	*This study*
MH1127*	*DF5, Mata/α YIplac211-MED11::URA3 euc1::natNT2 hub1::LEU2*	*This study*
MH1146*	*DF5, Mata/α YIplac211-MED11::URA3 euc1::natNT2 snt309::kanMX4*	*This study*
MH1129*	*DF5, Mata/α YIplac211-MED11::URA3 euc1::natNT2 bre1::kanMX4*	*This study*
MH1130*	*DF5, Mata/α YIplac211-MED11::URA3 euc1::natNT2 ubp8::hphNT1*	*This study*
MH1131*	*DF5, Mata/α YIplac211-MED11::URA3 euc1::natNT2 rad6::HIS3*	*This study*
MH1132*	*DF5, Mata/α YIplac211-MED11::URA3 euc1::natNT2 htb2::hphNT1 htb1-K123R::kanMX4*	*This study*
MH1227	*DF5, Mata YIplac211-MED11::URA3 mot3::kan*	*This study*
MH1228	*DF5, Mata YIplac211-MED11::URA3 euc1::natNT2 mot3::kan*	*This study*
MH1153*	*DF5, Mata/α YIplac211-MED11::URA3 euc1::natNT2 yef1::kanMX4*	*This study*
MH1154*	*DF5, Mata/α YIplac211-MED11::URA3 euc1::natNT2 rpl6b::kanMX4*	*This study*
MH1155*	*DF5, Mata/α YIplac211-MED11::URA3 euc1::natNT2 mco14::kanMX4*	*This study*
MH1176	*DF5, Mata YIplac211-MED11::URA3 nup133::hphNT1*	*This study*
MH1177	*DF5, Mata YIplac211-MED11::URA3 euc1::natNT2 nup133::hphNT1*	*This study*

MH063	*DF5, Mata pGPD-EUC1::natNT2*	*This study*
Y2726	*W303 (RAD5), Mata leu2-3,112 ade2-1 can1-100 his3-11,15 ura3-1 trp1-1*	*Xiaolan Zhao*
MH1125	*W303 (RAD5), Mata euc1::natNT2 leu2-3,112 ade2-1 can1-100 his3-11,15 ura3-1 trp1-1*	*This study*
MH1133	*W303 (RAD5), Mata euc1::natNT2 YIplac211-pGAL-empty-tADH::LEU2*	*This study*
MH1134	*W303 (RAD5), Mata euc1::natNT2 YIplac211-pEUC1-EUC1*	*This study*
MH1135	*W303 (RAD5), Mata euc1::natNT2 YIplac211-pGAL-EUC1-tADH*	*This study*
MH1136	*W303 (RAD5), Mata euc1::natNT2 YIplac211-pGAL-euc1-KR-tADH*	*This study*
MH1137	*W303 (RAD5), Mata euc1::natNT2 YIplac211-pGAL-euc1-SBM1+2-tADH*	*This study*
MH1138	*W303 (RAD5), Mata euc1::natNT2 YIplac211-pGAL-euc1-KR-SBM1+2-tADH*	*This study*
MH1139	*W303 (RAD5), Mata euc1::natNT2 YIplac211-pGAL-euc1-333WR>AA334-tADH*	*This study*
MH1140	*W303 (RAD5), Mata euc1::natNT2 YIplac211-pGAL-euc1-ΔN30-tADH*	*This study*
MH1141	*W303 (RAD5), Mata euc1::natNT2 YIplac211-pGAL-euc1-Δ104-129(F104>L)-tADH*	*This study*
MH1191	*DF5, Mata YIplac211-MED11::URA3 slx5::HIS3MX6*	*This study*
MH1192	*DF5, Mata YIplac211-MED11::URA3 sds3::kanMX4 slx5::HIS3MX6*	*This study*
MH1193	*DF5, Mata YIplac211-MED11::URA3 euc1::natNT2 slx5::HIS3MX6*	*This study*
MH1194	*DF5, Mata YIplac211-MED11::URA3 euc1::natNT2 sds3::kanMX4 slx5::HIS3MX6*	*This study*
MH417	*DF5, Mata/α Slx8::hphNT1 pGPD-EUC1::NatNT2*	*This study*
MH1239	*DF5, Mata/α slx5::HIS3MX4 pGPD-EUC1::natNT2*	*This study*
MH709	*YM4271, Mata YIplac211-3xub-HS4-pHIS3min-HIS3 euc1::kanMX4*	*This study*
MH1109	*DF5, Mata pADH-GFP-EUC1::NatNT2*	*This study*
MH1166	*DF5, Matα pADH-GFP-euc1-K231R-tADH::caURA3*	*This study*
MH1169	*DF5, Mata pADH-GFP-euc1-333WR>AA334-tADH::caURA3*	*This study*
MJK347	*W303 (RAD5), Mata YIplac211-pGAL-empty-tADH::LEU2*	*This study*
MJK534	*W303 (RAD5), Mata YIplac211-pGAL-EUC1-tADH*	*This study*
MH1199	*DF5, Mata YIplac211-MED11::URA3 slx8::hphNT1*	*This study*
MH1200	*DF5, Mata YIplac211-MED11::URA3 sds3::kanMX4 slx8::hphNT1*	*This study*
MH1201	*DF5, Mata YIplac211-MED11::URA3 euc1::natNT2 slx8::hphNT1*	*This study*
MH1202	*DF5, Matα YIplac211-MED11::URA3 euc1::natNT2 sds3::kanMX4 slx8::hphNT1*	*This study*

Appendix Table S2. Plasmids used in this study.

Position for all protein truncation constructs refer to amino acid (aa) positions in WT proteins.

Plasmid	Construct	Ref./Source
pMax114	*YIplac128-Hotspot 5*	This study
pMax115	*YIplac128-Hotspot 5-F1*	This study
pMax116	*YIplac128-Hotspot 5-F2*	This study
pMax117	*YIplac128-Hotspot 5-F3*	This study
pMax118	*YIplac128-Hotspot 5-F4*	This study
pMax125	*YIplac128-Hotspot 5-F5*	This study
pMax135	*YIplac128-Hotspot 5-F6*	This study
pMax144	*YIplac128-Hotspot 5-F7*	This study
pMax145	*YIplac128-Hotspot 5-F7-Mut*	This study
pMax138	*YIplac128-Hotspot 5-F8*	This study
pMax197	*YIplac211-min.promotor-HIS3*	This study
pMax193	*YIplac211-3xHotspot 5-F7-min.promotor-HIS3*	This study
pMax196	*YIplac211-3xHotspot 5-F7-Mut-min.promoter-HIS3*	This study
pMax218	*pGAD-HA-5'UTR-SUMO*	This study (Y1H screen)
pMax219	*pGAD-HA-YFR006W-38-535*	This study (Y1H screen)
pMax223	*pGAD-HA-EUC1-174-462*	This study (Y1H screen)
pGAD-C1	*Gal4-AD*	(James *et al*, 1996)
pGBD-C1	*Gal4-BD*	(James *et al*, 1996)
pMax209	*pGAD-EUC1*	This study
pMax198	*PGBD-EUC1*	This study
pMax242	*pGAD-euc1-K231R*	This study
D1097	*pGAD424-SUMO-GG*	Jentsch DNA collection
D1099	*pGAD424-SUMO-AA*	Jentsch DNA collection
pMax90	*pGAD-UBC9*	This study
D1674	*pGAD-SIZ1*	Jentsch DNA collection
pMax122	*pGAD-SIZ2*	This study
pMH237	*pGAD-EUC1-aa16-end*	This study
pMH238	*pGAD-EUC1-aa31-end*	This study
p415-ADH	*p415-pADH-empty*	(Mumberg *et al*, 1995)
pMH281	*p415-ADH-3FLAG-EUC1*	This study
pMH282	*p415-ADH-3FLAG-EUC1-K231R*	This study
pMH120	*pGAD-EUC1-81-183*	This study
pMH53	*pGBD-SLX5*	This study
pMH55	*pGBD-SLX5-1-487 (ΔRING)*	This study
pMH11	*p415-pEUC1-empty*	This study
pMH197	*pEUC1-3FLAG-EUC1*	This study
pMH198	*pEUC1-3FLAG-EUC1-KR*	This study
pMH217	*pEUC1-3FLAG-EUC1-W333A,R334A (DBD*)*	This study
pMH294	*pEUC1-3FLAG-EUC1-SBM1_mut*	This study

pMH295	*pEUC1-3FLAG-EUC1-SBM2_mut*	This study
pMH296	*pEUC1-3FLAG-EUC1-SBM1+2_mut*	This study
pMH300	*pEUC1-3FLAG-EUC1-KR-SBM1_mut*	This study
pMH301	*pEUC1-3FLAG-EUC1-KR-SBM2_mut*	This study
pMH302	*pEUC1-3FLAG-EUC1-KR-SBM1+2_mut*	This study
pMH124	*pGAD-EUC1-81-183_SBM1_mut*	This study
pMH125	*pGAD-EUC1-81-183_SBM2_mut*	This study
pMH135	*pGAD-EUC1-81-183_SBM1+2_mut*	This study
pMH57	*pGBD-SLX5-1-337*	This study
pMH264	*pGBD-SLX5-201-335 (Md)*	This study
pMH266	*pGBD-SLX5-1-200,339-end (MdΔ)*	This study
pMH318	*p415-pADH-3FLAG-EUC1-SBM1_mut*	This study
pMH319	*p415-pADH-3FLAG-EUC1-SBM2_mut*	This study
pMH320	*p415-pADH-3FLAG-EUC1-SBM1+2_mut*	This study
pMH321	*p415-pADH-3FLAG-EUC1-KR-SBM1_mut*	This study
pMH322	*p415-pADH-3FLAG-EUC1-KR-SBM2_mut*	This study
pMH323	*p415-pADH-3FLAG-EUC1-KR-SBM1+2_mut*	This study
p414-ADH	*p414-pADH-empty*	(Mumberg *et al*, 1995)
pMH326	*p414-pSLX5-NLS-3HA-SLX5*	This study
pMH327	*p414-pSLX5-NLS-3HA-SLX5_SIM1-5mut (SIM*)*	This study
pMH330	*p414-pSLX5-NLS-3HA-SLX5_1-200_339-end (MdΔ)*	This study
pMH146	*pGAD-EUC1-aa240-462*	This study
pMH147	*pGAD-EUC1-aa291-462*	This study
pMH148	*pGAD-EUC1-aa291-335*	This study
pMH149	*pGAD-EUC1-aa291-385*	This study
pMH150	*pGAD-EUC1-aa291-425*	This study
pMH151	*pGAD-EUC1-aa336-462*	This study
pMH152	*pGAD-EUC1-aa386-462*	This study
pMH153	*pGAD-EUC1-aa426-462*	This study
pMH157	*pGAD-EUC1-FL_W333A,R334A (DBD*)*	This study
D2431	*YEplac195-pADH-BD-tADH*	(Moldovan *et al*, 2006)
pMH273	*YEplac195-pADH-EUC1-1-30-HA-BD-tADH*	This study
pMH268	*YEplac195-pADH-EUC1-1-295-HA-BD-tADH*	This study
pMH269	*YEplac195-pADH-EUC1-1-295KR-HA-BD-tADH*	This study
pMH63	*pGAD-EUC1-AA1-220*	This study
pMH77	*pGAD-EUC1-1-183*	This study
pMH78	*pGAD-EUC1-1-140*	This study
pMH79	*pGAD-EUC1-1-100*	This study
pMH64	*pGAD-EUC1-1-60*	This study
pMH68	*pGAD-EUC1-161-220*	This study
pMH82	*pGAD-EUC1-121-220*	This study
pMH81	*pGAD-EUC1-81-220*	This study
pMH80	*pGAD-EUC1-41-220*	This study
pMH222	*pGBD-EUC1-81-183*	This study
pMH261	*pGAD-EUC1_Δ104-129 (F104>L), (CCΔ)*	This study

pMH222	*pGBD-EUC1-81-183*	This study
pMH223	*pGBD-EUC1-81-183_SBM1_mut*	This study
pMH224	*pGBD-EUC1-81-183_SBM2_mut*	This study
pMH225	*pGBD-EUC1-81-183_SBM1+2_mut*	This study
pMH249	*pGBD-SLX5-aa1-275*	This study
pMH250	*pGBD-SLX5-aa1-200*	This study
pMH251	*pGBD-SLX5-aa1-145*	This study
pMH252	*pGBD-SLX5-aa1-90*	This study
pMH253	*pGBD-SLX5-aa1-50*	This study
pMH324	*p414-pSLX5-NLS-3HA*	This study
pMH333	*p414-pSLX5-3HA-SLX5*	This study
pMH334	*p414-pSLX5-3HA-SLX5_SIM1-5mut (SIM*)*	This study
pMH337	*p414-pSLX5-3HA-SLX5_1-200_339-end (MdΔ)*	This study
pMH14	*p415-pEUC1-EUC1*	This study
pMH291	*p415-pEUC1-EUC1-SBM1_mut*	This study
pMH292	*p415-pEUC1-EUC1-SBM2_mut*	This study
pMH293	*p415-pEUC1-EUC1-SBM1+2_mut*	This study
pMH368	*p415-pGAD-C1-EUC1-SBM1_mut*	This study
pMH369	*p415-pGAD-C1-EUC1-SBM2_mut*	This study
pMH370	*p415-pGAD-C1-EUC1-SBM1+2_mut*	This study
pMH365	*p415-pMET25-a2(103-189)-URA3-3HA-6His*	(Hickey & Hochstrasser, 2015)
pMH190	*YCplac111-MED11 (479us-307ds)*	This study
pMH191	*YCplac111-EUC1 (403us-328ds)*	This study
pMH447	*p413-pADH-SLX5-tCYC1*	This study
pMH218	*p414-pADH-SLX8-tADH*	This study
pMH15	*p415-pEUC1-euc1-K231R*	This study
pMH299	*p415-pEUC1-euc1-K231R-SBM1+2**	This study
pMH367	*p415-pEUC1-euc1-W333A,R334A (DBD*)*	This study
pMH244	*p415-pEUC1-euc1-aa31-end (N30Δ)*	This study
pMH260	*p415-pEUC1-euc1-Δ104-129 (F104>L) (CCΔ)*	This study

Appendix Table S3. Primers used for qPCR.

Name	Position	Sequence
ub-HS1_F	ChrIII_123537	TTTCTGCCAGTAGCGACACCACACAT
ub-HS1_R	ChrIII_123719	ATGACGATGGCAGGGAAAATAGGGCTGT
ub-HS1_motif1_F	ChrIII_123811	GTAACCCTGCGTCACACATGAGAA
ub-HS1_motif1_R	ChrIII_123985	TCACAGTTTACCCGGAGGTCATCA
ub-HS1_motif2_F	ChrIII_125415	TGTTTTATGCGGAAATTGCAGTGGA
ub-HS1_motif2_R	ChrIII_125547	ATGTATGGTTAAGCAGGCTTTGCG
ub-HS2_F	ChrIV_358238	CCTTGTCAGATAATGTATGGGTGGTGTG
ub-HS2_R	ChrIV_358367	TATTCTTTGTGTTCGCATTCGCTTCCC
ub-HS3_F	ChrIV_1087121	AACAATAGAAAAACGCGGGACTCGAT
ub-HS3_R	ChrIV_1087280	TGCTAATTTTCAGCCACATCACATGC
ub-HS4_F	ChrXIII_309445	TGGAAGCATCACATCGTATGCTACTAGA
ub-HS4_R	ChrXIII_309647	TATGTATGCGGCAATGAACTACTCCGA
ub-HS4_motif1+2_F	ChrXIII_308867	TAAAGTGCATTCAAACATCGGCAGG
ub-HS4_motif1+2_R	ChrXIII_309021	CCCACGACAGCGGTATCTATCTTT
ub-HS4_motif3_F	ChrXIII_309619	TTTCGGAGTAGTTCATTGCCGCAT
ub-HS4_motif3_R	ChrXIII_309760	ACGCATCCATGTCGTGTACATTTC
ub-HS5_A_F	ChrXIII_413843	AACGACGTACCCACTACGCGTTTGAA
ub-HS5_A_R	ChrXIII_414033	AACTGTTGGAATGTGAGGGCGACCTAGT
ub-HS5_B_F	ChrXIII_413474	ATCTGAGCACACACTTCCTCCTGA
ub-HS5_B_R	ChrXIII_413659	GAAATCCTAGCTGCGAACGGGAAA
ub-HS6_F	ChrXIII_433645	TCTTTGCACAATGCATTACGTGGGAG
ub-HS6_R	ChrXIII_433789	GAGAAATAGATTCAATGCCGTGGCGA
ub-HS7_F	ChrXV_168011	TGTTACGCGTTCCATTTGAGAAGCAA
ub-HS7_R	ChrXV_168209	CGGCTTTAAACACCCGTGCCTATATT
contr_F	ChrII_564535	ACCGACTAATGCGGTCATGGAAAGC
contr_R	ChrII_564727	CTTTTCTCGCAAGAAGACTCCAGAATCA
ect-ub-HS_F	YI128 backbone	CATTAATGCAGCTGGCACGACAGGTT
ect-ub-HS_R	YI128 backbone	ACAATTCCACACAACATACGAGCCGGA
Act1_F	ORF	GAAATGCAAACCGCTGCTCAATCTTC
Act1_R	ORF	CAATACCGGCAGATTCCAAACCCAAA
HIS3_F	ORF	TTACCCTCCACGTTGATTGTCTGC
HIS3_R	ORF	AACACCTTTGGTGGAGGGAACATC
ub-only-site1_F	ChrIV_1117000	GCATCTATCGTATTCTTGAGTTATTGCGAC
ub-only-site1_R	ChrIV_1117000	ATGTCAATACCATCAGGATCTTGCATGA
STI1-CIN5_F	ChrXV_382999	GGACCATCTTTCCTGTCGTTCTCC
STI1-CIN5_r	ChrXV_383175	GCTTAGCGAATGTTGTCATGGAGC
TEC1-us_F	ChrII_408761	TGAATTCGGGAATGTGCGTGTTTC
TEC1-us_R	ChrII_408921	AGCACCATGGATTGCTGATGGTAG
PGK1_F	ORF	GTAAGGCTTTGGAGAACCCAACCA
PGK1_R	ORF	TGAAGGTGAAAGCCATACCACCAC
ILV5_F	ORF	AACTCTTCTTACGCCGTCTGGAAC
ILV5_R	ORF	AGAACATACCGTGGATACCACCCA

SSF2_F	ORF	GGCTGTTAAAGATGCTAAAAAGCAACG
SSF2_R	ORF	GAAGATCCATCATCGCTCATTGCAC
EUC1_F	ORF	CCGTCAGTTCTTTCCCTTGAGAGG
EUC1_R	ORF	CGACAACCTTGATGGCTTGGTTTC
HSP12_F	ORF	TGTCCACGACTCTGCCGAAA
HSP12_R	ORF	CAACTTGGACTTGGCGGCTC
SIR2_F	ORF	CCTTCCCACGTTCCCCAAGT
SIR2_R	ORF	TATGCGGAATCGTCCAGCCA
SBH2_F	ORF	AGTTCCACCAGGAGGTCAGC
SBH2_R	ORF	AAGACCCACCGTAACCAGCC
RCO1_F	ORF	CCCAAAATGGCAATAGCGAGGA
RCO1_R	ORF	GTTCGTTCGGCACGACTACG
PFK1_F	ORF	ACTGCTATCCCAGGCCATGT
PFK1_R	ORF	AGCGTCAGTGTTTGGAGAAGC
ALD5_F	ORF	GGGCTCGTCTTGTGACTGGA
ALD5_R	ORF	TGGGACCAAACACTTCCTCCT
CIN5_F	ORF	TGCAAGGCCGGTGACAATAA
CIN5_R	ORF	ATGAAGCTGCCGGTTGGCTA
MRH1_F	ORF	CGGTGCTGACAAATTGGGCT
MRH1_R	ORF	TGGTGTAGCAGCAGGTCTTGG
ADH2_F	ORF	CGTTAAGGCTACCAACGGCG
ADH2_R	ORF	TTCCCCACGTAAGAGCCGAC
CDC19_F	ORF	TTACAACCCAAGACCAACCAGAGC
CDC19_R	ORF	CTTGTTCAGCAATGACAGCGGTTT

Appendix References

Aparicio O, Geisberg JV, Sekinger E, Yang A, Moqtaderi Z & Struhl K (2005) Chromatin immunoprecipitation for determining the association of proteins with specific genomic sequences in vivo. *Curr Protoc Mol Biol* **Chapter 21:** Unit 21.3

Bailey TL, Boden M, Buske FA, Frith M, Grant CE, Clementi L, Ren J, Li WW & Noble WS (2009) MEME SUITE: tools for motif discovery and searching. *Nucleic Acids Res* **37:** W202–W208

Böhm S & Buchberger A (2013) The budding yeast Cdc48(Shp1) complex promotes cell cycle progression by positive regulation of protein phosphatase 1 (Glc7). *PLoS ONE* **8:** e56486

Byrne KP & Wolfe KH (2005) The Yeast Gene Order Browser: combining curated homology and syntenic context reveals gene fate in polyploid species. *Genome Res* **15:** 1456–1461

Chen P, Johnson P, Sommer T, Jentsch S & Hochstrasser M (1993) Multiple ubiquitin-conjugating enzymes participate in the in vivo degradation of the yeast MAT alpha 2 repressor. *Cell* **74:** 357–369

Durinck S, Moreau Y, Kasprzyk A, Davis S, De Moor B, Brazma A & Huber W (2005) BioMart and Bioconductor: a powerful link between biological databases and microarray data analysis. *Bioinformatics* **21:** 3439–3440

Durinck S, Spellman PT, Birney E & Huber W (2009) Mapping identifiers for the integration of genomic datasets with the R/Bioconductor package biomaRt. *Nat Protoc* **4:** 1184–1191

Finley D, Ozkaynak E & Varshavsky A (1987) The yeast polyubiquitin gene is essential for resistance to high temperatures, starvation, and other stresses. *Cell* **48:** 1035–1046

Gautier L, Cope L, Bolstad BM & Irizarry RA (2004) affy--analysis of Affymetrix GeneChip data at the probe level. *Bioinformatics* **20:** 307–315

Gentleman RC, Carey VJ, Bates DM, Bolstad B, Dettling M, Dudoit S, Ellis B, Gautier L, Ge Y, Gentry J, Hornik K, Hothorn T, Huber W, Iacus S, Irizarry R, Leisch F, Li C, Maechler M, Rossini AJ, Sawitzki G, et al (2004) Bioconductor: open software development for computational biology and bioinformatics. *Genome Biol.* **5:** R80

Hickey CM & Hochstrasser M (2015) STUbL-mediated degradation of the transcription factor MATα2 requires degradation elements that coincide with corepressor binding sites. *Mol Biol Cell* **26:** 3401–3412

Hoege C, Pfander B, Moldovan G-L, Pyrowolakis G & Jentsch S (2002) RAD6-dependent DNA repair is linked to modification of PCNA by ubiquitin and SUMO. *Nature* **419:** 135–141

Irizarry RA, Hobbs B, Collin F, Beazer-Barclay YD, Antonellis KJ, Scherf U & Speed TP (2003) Exploration, normalization, and summaries of high density oligonucleotide array probe level data. *Biostatistics* **4:** 249–264

James P, Halladay J & Craig EA (1996) Genomic libraries and a host strain designed for highly efficient two-hybrid selection in yeast. *Genetics* **144:** 1425–1436

Janke C, Magiera MM, Rathfelder N, Taxis C, Reber S, Maekawa H, Moreno-Borchart A, Doenges G, Schwob E, Schiebel E & Knop M (2004) A versatile toolbox for PCR-based tagging of yeast genes: new fluorescent proteins, more markers and promoter substitution cassettes. *Yeast* **21:** 947–962

Kalocsay M, Hiller NJ & Jentsch S (2009) Chromosome-wide Rad51 spreading and SUMO-H2A.Z-dependent chromosome fixation in response to a persistent DNA double-strand break. *Mol Cell* **33:** 335–343

Kelley LA, Mezulis S, Yates CM, Wass MN & Sternberg MJE (2015) The Phyre2 web portal for protein modeling, prediction and analysis. *Nat Protoc* **10:** 845–858

Knop M, Siegers K, Pereira G, Zachariae W, Winsor B, Nasmyth K & Schiebel E (1999) Epitope tagging of yeast genes using a PCR-based strategy: more tags and improved practical routines. *Yeast* **15:** 963–972

Koegl M, Hoppe T, Schlenker S, Ulrich HD, Mayer TU & Jentsch S (1999) A novel ubiquitination factor, E4, is involved in multiubiquitin chain assembly. *Cell* **96:** 635–644

Moldovan G-L, Pfander B & Jentsch S (2006) PCNA controls establishment of sister chromatid cohesion during S phase. *Mol Cell* **23:** 723–732

Mumberg D, Müller R & Funk M (1995) Yeast vectors for the controlled expression of heterologous proteins in different genetic backgrounds. *Gene* **156:** 119–122

O'Geen H, Nicolet CM, Blahnik K, Green R & Farnham PJ (2006) Comparison of sample preparation methods for ChIP-chip assays. *Biotechniques* **41:** 577–580

Prytuliak R, Volkmer M, Meier M & Habermann BH (2017) HH-MOTiF: de novo detection of short linear motifs in proteins by Hidden Markov Model comparisons. *Nucleic Acids Res* **45:** W470–W477

Rape M, Hoppe T, Gorr I, Kalocay M, Richly H & Jentsch S (2001) Mobilization of processed, membrane-tethered SPT23 transcription factor by CDC48(UFD1/NPL4), a ubiquitin-selective chaperone. *Cell* **107:** 667–677

Renkawitz J, Lademann CA, Kalocsay M & Jentsch S (2013) Monitoring homology search during DNA double-strand break repair in vivo. *Mol Cell* **50:** 261–272

Richly H, Rape M, Braun S, Rumpf S, Hoege C & Jentsch S (2005) A series of ubiquitin binding factors connects CDC48/p97 to substrate multiubiquitylation and proteasomal targeting. *Cell* **120:** 73–84

Rumpf S & Jentsch S (2006) Functional Division of Substrate Processing Cofactors of the Ubiquitin-Selective Cdc48 Chaperone. *Mol Cell* **21:** 261–269

van de Pasch LAL, Miles AJ, Nijenhuis W, Brabers NACH, van Leenen D, Lijnzaad P, Brown MK, Ouellet J, Barral Y, Kops GJPL & Holstege FCP (2013) Centromere binding and a conserved role in chromosome stability for SUMO-dependent ubiquitin ligases.

PLoS ONE **8:** e65628

Wilson CL & Miller CJ (2005) Simpleaffy: a BioConductor package for Affymetrix Quality Control and data analysis. *Bioinformatics* **21:** 3683–3685

Xie Y, Kerscher O, Kroetz MB, McConchie HF, Sung P & Hochstrasser M (2007) The yeast Hex3.Slx8 heterodimer is a ubiquitin ligase stimulated by substrate sumoylation. *J Biol Chem* **282:** 34176–34184

Yang L, Mullen JR & Brill SJ (2006) Purification of the yeast Slx5-Slx8 protein complex and characterization of its DNA-binding activity. *Nucleic Acids Res* **34:** 5541–5551
