# Supplementary material for: Slx5/Slx8‐dependent ubiquitin hotspots on chromatin contribute to stress tolerance
Source: EMBO J. 2019 Apr 23;38(11):e100368. doi: 10.15252/embj.2018100368 (PMC6545562; doi:10.15252/embj.2018100368)
Supplement: Supplementary file 2 — Expanded View Figures PDF [file EMBJ-38-e100368-s002.pdf]

Expanded View Figures

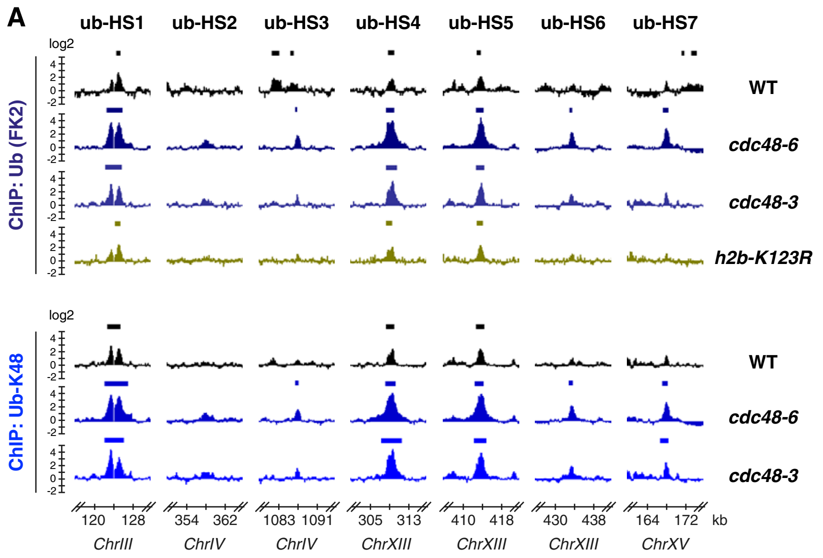

B

Region	Chrom.	Start	End
ub-HS1	III	122959	126313
ub-HS2	IV	357421	359358
ub-HS3	IV	1086796	1087308
ub-HS4	XIII	308624	310463
ub-HS5	XIII	412924	414521
ub-HS6	XIII	433248	433923
ub-HS7	XV	167527	168701

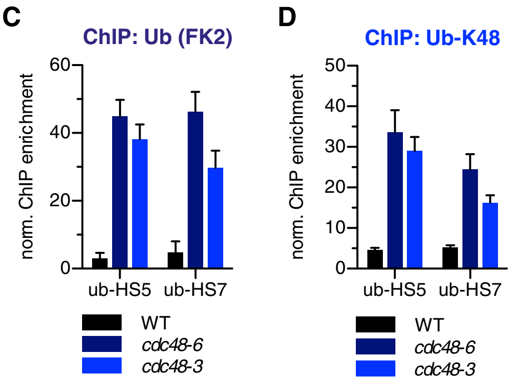

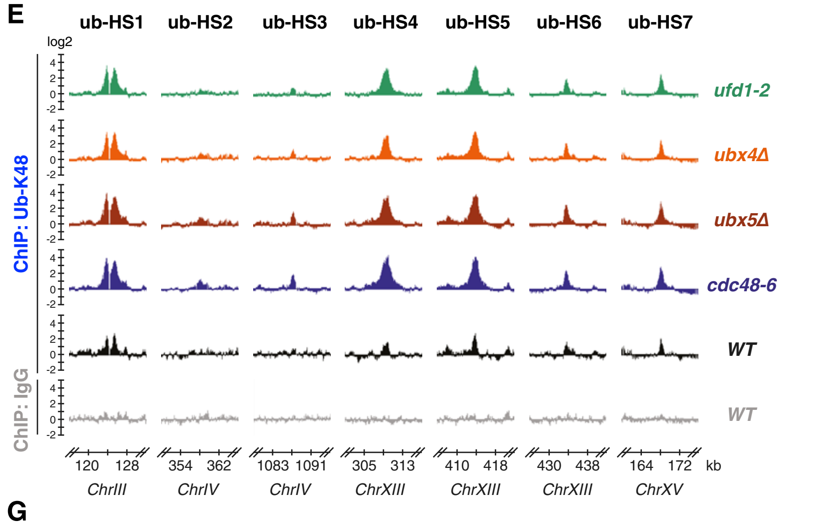

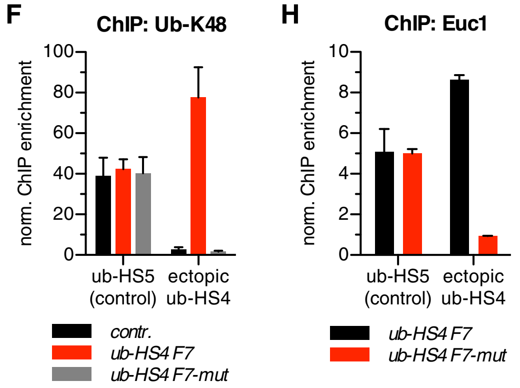

G

Gene	Protein start site in aa (number of clones)	Clones screen 1 (*ubx5Δ*)	Clones screen 2 (WT)	tot. clones in frame
SMT3 (SUMO)	several 5'UTR positions	-	18	18
SLX5	152 (1x), 153 (9x), 197 (1x), 218 (1x), N.D. (4x)	16	-	11
YMR111C/EUC1	38 (3x), 174 (1x)	3	1	4
YFR006W	38 (4x)	-	4	4

Ectopic *(ChrIII)* fragments of ub-HS4

ub-HS4 F7 CCCAGTTGTTACGTATTTGTTTCCCGTTCCATTCATGAT
ub-HS4 F7-mut CCCAGTTGTTACGTATTTTTTTCCCGTTCCATTCATGAT

Figure EV1.

© 2019 The Authors

Figure EV1. Related to Figs 1 and 2. Seven ubiquitin hotspots across the yeast genome share a Cdc48-dependent extraction mechanism and recruit Ymr111c/ Euc1.

A The FK2 ubiquitin antibody and a ub-K48 specific antibody detect the same ub-HSs in genome-wide ChIP-chip experiments. 16-kb windows from genome-wide ChIP-chip data using either the ubiquitin (FK2) or ub-K48 (clone Apu2) antibodies are shown. For comparison, data for ubiquitin (FK2) ChIP in WT, *cdc48-6*, *cdc48-3* are reproduced from Fig 1B and for *h2b-K123R* partially (ub-HS4) from Fig 1A. Data represent means from two independent replicates.

B Table summarizing the ub-HSs identified in Fig 1B. Stretches were defined by significantly enriched regions in ub-K48 ChIP-chip in *cdc48-6* (all except ub-HS2) or Slx8-9myc-enriched regions in the *cdc48-3* background (ub-HS2).

C, D Ubiquitin signals increase around 5–10-fold in both *cdc48-6* and *cdc48-3* mutants with ubiquitin (FK2) and ub-K48-chain-specific antibodies. ChIP-qPCR experiment using ubiquitin (FK2) (C) and ub-K48 (D) antibodies and indicated strains. Data represent means ± SD (n = 3 for (C), n = 4 for (D)).

E *ufd1-2*, *ubx4*Δ, and *ubx5*Δ show an increase of ubiquitin conjugates at all ub-HSs similar to *cdc48-6*. Genome-wide ChIP-chip data using the ub-K48 or a non-specific IgG control antibody for the indicated strains. Data for WT and *cdc48-6* reproduced from (A) for comparison. Data represent means from two independent replicates.

F A single point mutation within the ub-HS-motif abolishes ubiquitin enrichment. A G>T mutation was introduced in one of the conserved TTGTT repeats of *ub-HS4 F7* (bottom scheme) and integrated at the *LEU2* locus as described in Fig 2A. ChIP-qPCR for ub-K48 demonstrated that the ubiquitin enrichment is lost upon mutation of the ub-HS-motif (*ub-HS4 F7-mut*). Experiments were performed in *cdc48-6* strains. Data represent means ± SD (n = 5).

G Table summarizing the confirmed hits from two independent Y1H screens as described in Fig 2D. Protein start sites are indicated. aa: amino acid, N.D.: not determined.

H Endogenous Euc1 does not bind the mutated ub-HS4-motif. ChIP with an Euc1-specific antibody was performed in strains with *ub-HS4 F7* or *ub-HS4 F7-mut* integrated at the *LEU2*-locus as described in (F). Experiments were performed in *cdc48-6* strains. Data represent means ± SD (n = 2).

Figure EV2. Related to Fig 4. Slx5/Slx8-mediated ubiquitylation does not lead to fast Euc1 degradation.

A Euc1 binding to ub-HSs drops in *euc1-KR* cells. ChIP against Euc1 was quantified by qPCR. Data represent means ± SD (n = 2).

B Slx5 is required for Euc1 ubiquitylation. Denaturing NiNTA-PDs with ^{His}Ubi as in Fig 4E. Note that Euc1 ubiquitylation levels were abolished in *slx5*Δ cells, but also reduced in *cdc48-3* cells.

C, D Euc1 levels increase in *slx5*Δ and for *euc1-KR*. Euc1 levels were quantified from WBs of three replicate samples (C) using a LI-COR Odyssey Fc imaging system and normalized to Pgk1 and wild-type levels (D). Pgk1 served as loading control. Data represent means ± SD (n = 3).

E Euc1 shows slow degradation kinetics. Cells were treated with 0.5 mg/ml cycloheximide (CHX), and samples were taken at the indicated times. Quantification was done as in (C–D). Relative Euc1 signals are normalized to Dpm1 levels and to t = 0.

F Euc1 and ub-K48 ChIP signals do not correlate. ChIP against Euc1 (top) and ub-K48 (bottom) analyzed by qPCR for all ub-HSs. Separate primer pairs were used for distinct motif occurrences within ub-HS1 and ub-HS4. Note that Euc1 signals did not increase in a *cdc48-3* strain. Data for ub-K48 ChIP for ub-HS3/HS5/HS7 are reproduced from Fig 1E for comparison. Data represent means ± SD (n = 2).

G Euc1 binding to ub-HS sites is reduced—rather than increased—in *slx5*Δ and *slx8*Δ cells. ChIP against Euc1 was quantified by qPCR in the indicated strains. Note that the binding defect for Euc1 is similar for the *euc1-KR* and *slx5*Δ strains. Data represent means ± SD (n = 2).

H SUMOylation is required for recruitment of Slx8-9myc to ub-HSs. ChIP against Slx8-9myc was quantified by qPCR. Data represent means ± SD (n = 3).

I Genome-wide binding profiles of Slx8 in *euc1*Δ and *ubc9-1* mutant cells. ChIP-chip was performed as described in Fig 1A. Binding profiles for ub-K48 (*cdc48-3*) and for Slx8-9myc (WT, *cdc48-3*) are reproduced for comparison from Figs EV1A and 1B, respectively. Data represent means from two independent replicates.

Source data are available online for this figure.

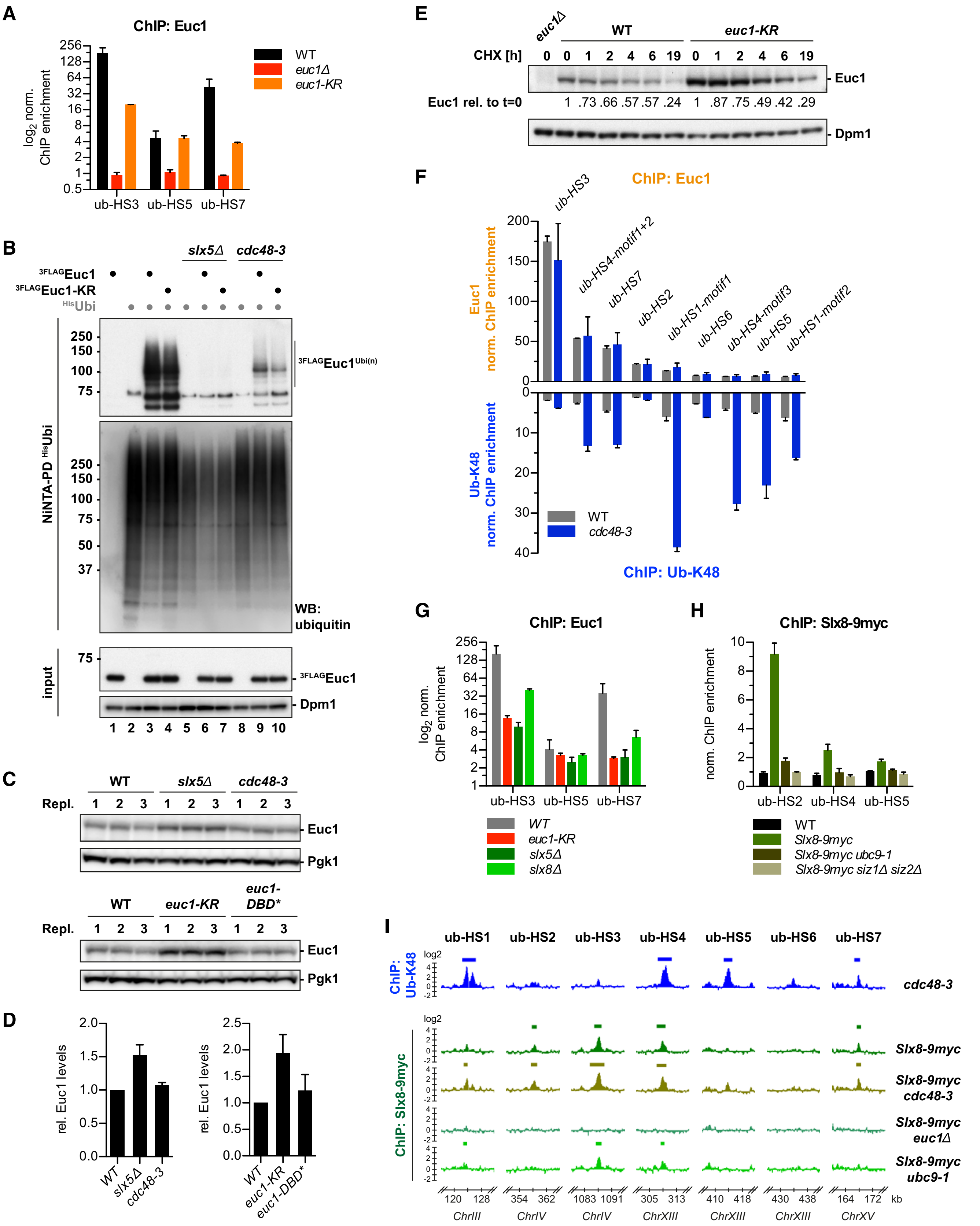

Figure EV2.

© 2019 The Authors

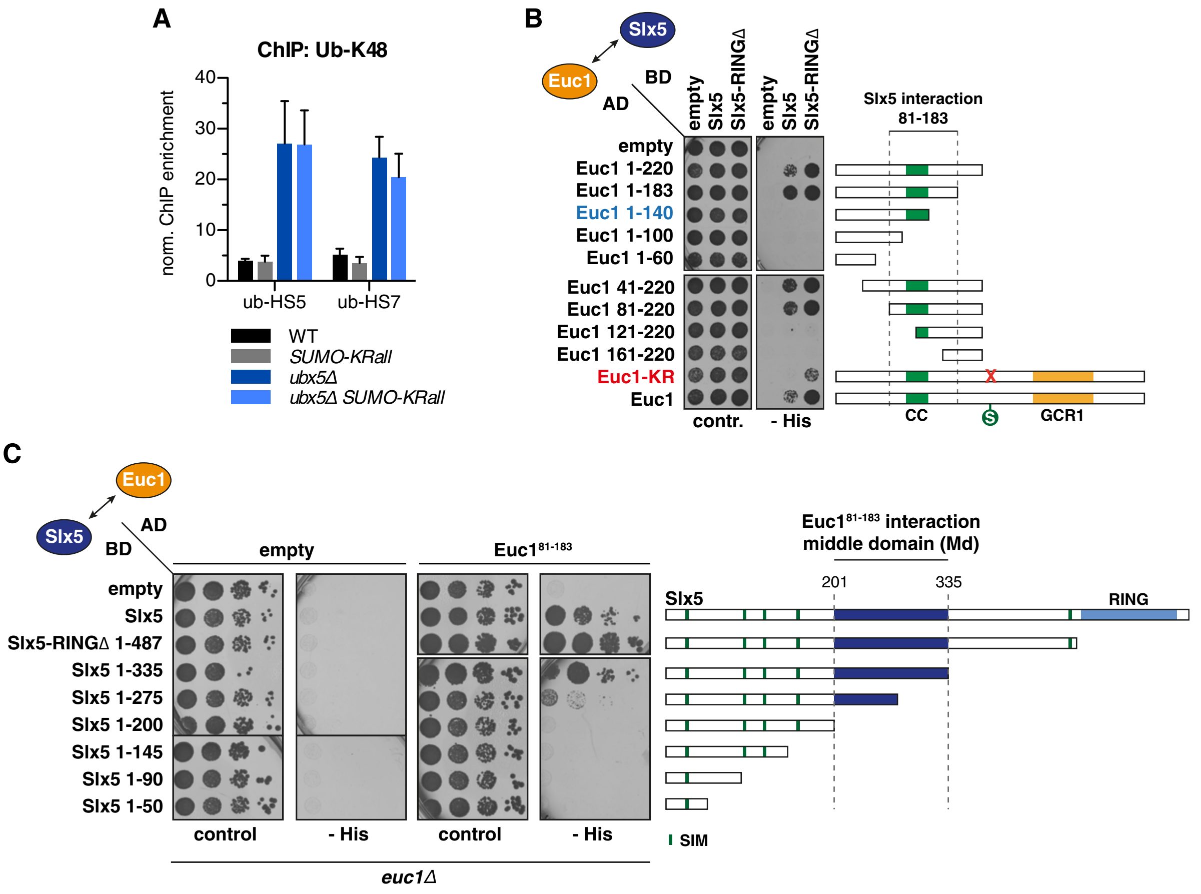

Figure EV3. Related to Fig 5. Euc1$^{81–183}$ interacts with the Slx5 middle domain (Slx5-Md, aa 201–335).†

A SUMO-chain formation is not required for ub-HS formation. ChIP against ub-K48 in strains expressing a SUMO variant with all lysines mutated to arginines (*SUMO-KRall*) as the only source of SUMO. Enriched DNA was analyzed by qPCR. Data represent means ± SD (n = 3).

B The region of Euc1 required for interaction with Slx5 maps to aa 81–183. Y2H assay to map the Slx5 interaction site on Euc1. Note that SUMOylation-deficient Euc1-KR still interacts with Slx5-RINGΔ, albeit weaker than wild-type Euc1 (bottom 2 rows). Cells were grown at 30°C for 2 days.

C The region of Slx5 required to interact with Euc1 maps to aa 201–335. C-terminal Gal4-BD-Slx5 truncation constructs were probed for interaction with Euc1$^{81–183}$ in Y2H. Note that the interaction gradually decreases when truncations between aa 201 and 487 were made. We defined aa 201–335 (middle domain, Slx5-Md) to be the minimal region required for robust interaction with Euc1 (Fig 5F); however, the region between aa 336 and 487 also contributes to the interaction (compare Slx5-RINGΔ and Slx5-Md in Fig 5D and G). Cells were grown at 30°C for 3 days.

†Correction added on 29 April 2019 after first online publication: Slx5-RING was corrected to Slx5-RINGΔ in panels B and C.

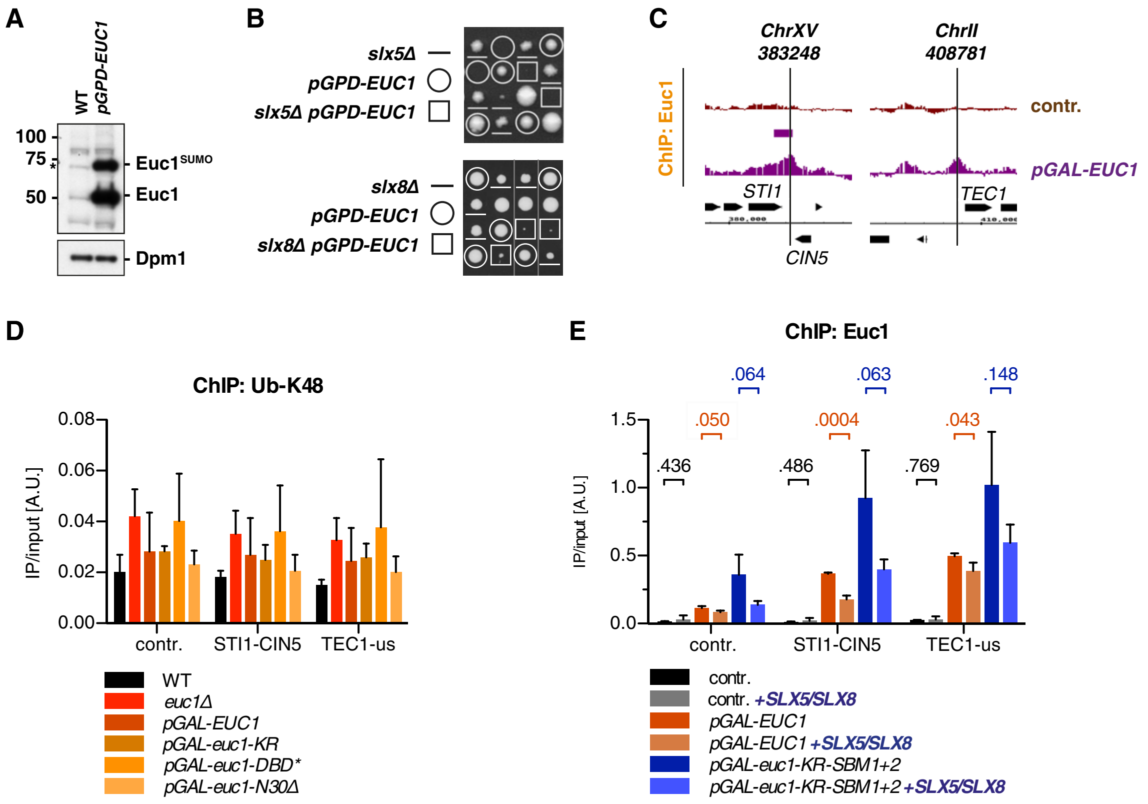

Figure EV4. Related to Fig 7. Slx5/Slx8 alleviates *EUC1* overexpression toxicity by curbing ectopic Euc1 localization.

A Western blot against Euc1 (and Dpm1 as loading control) to compare expression levels in WT and *pGPD-EUC1* cells. Asterisk denotes a non-specific band.

B *EUC1* overexpression leads to strong phenotypes or lethality in *slx5*Δ and *slx8*Δ cells. Individual cells from tetrads (arranged in vertical columns) were grown on YPD plates at 30°C for 3 days.

C ChIP-chip tracks from regions showing additional *EUC1*-binding signals upon *EUC1*-overexpression (*pGAL-EUC1*, 3 h induction). Data represent means from two independent replicates.

D Ubiquitin (ub-K48) ChIP-qPCR for the same samples as shown in Fig 7E. Data represent means ± SD (*n* = 3). A.U.: arbitrary units.

E Overexpression of *SLX5/SLX8* leads to a reduction of aberrant Euc1 binding to non-ub-HS loci. ChIP-qPCR analysis of selected strains from Fig 7F as indicated, after 3-h galactose induction. Data represent means ± SD (*n* = 3). *P*-values from Student's *t*-tests for the indicated comparisons are shown.

 © 2019 The Authors
