# Supplementary figures and images for: Slx5/Slx8‐dependent ubiquitin hotspots on chromatin contribute to stress tolerance
Source: EMBO J. 2019 Apr 23;38(11):e100368. doi: 10.15252/embj.2018100368 (PMC6545562; doi:10.15252/embj.2018100368)

Source data for Figure 4B.

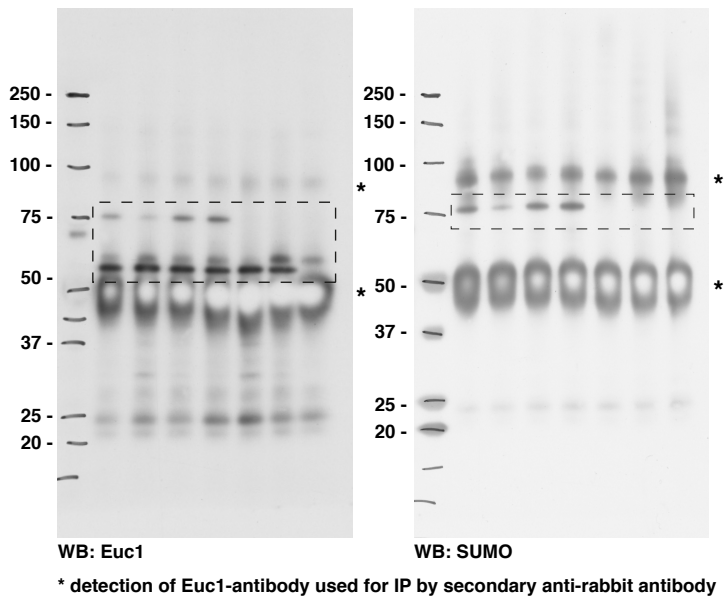

Source data for Figure 4C.

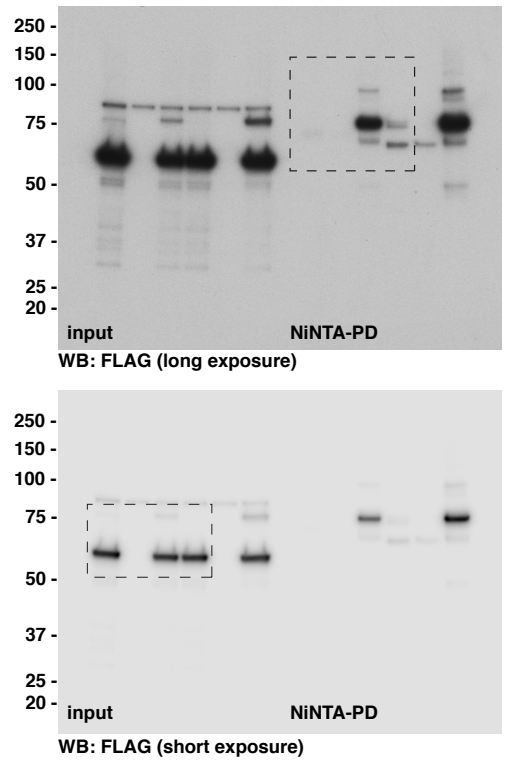

Source data for Figure 4E.

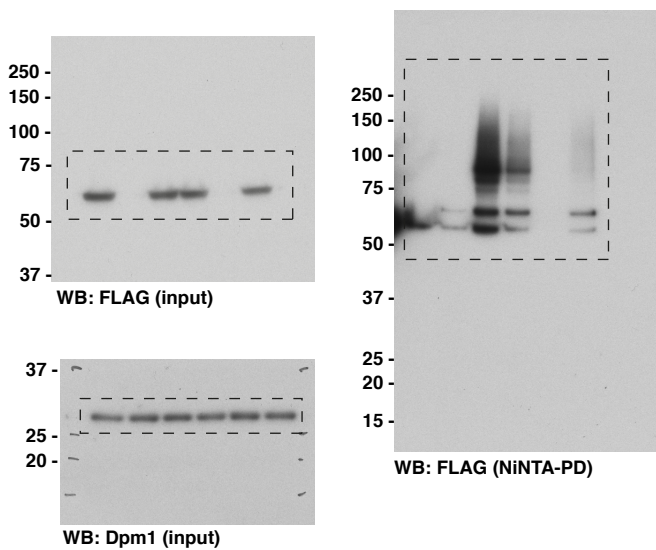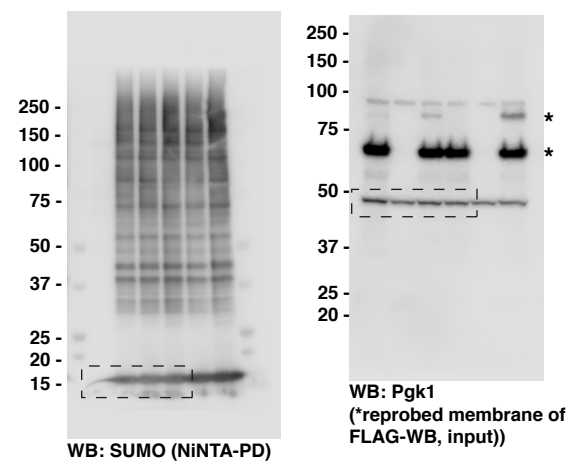

Supplement: Supplementary file 14 — Source Data for Figure 4 [file EMBJ-38-e100368-s012.pdf]

Source Data for Figure 5B.

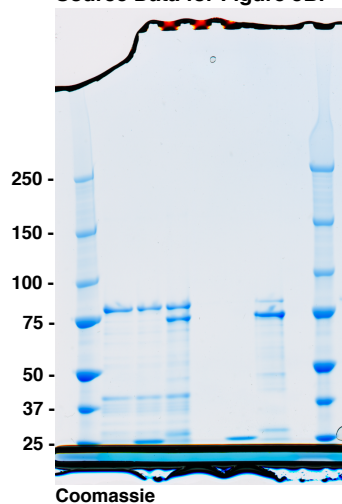

Source Data for Figure 5C.

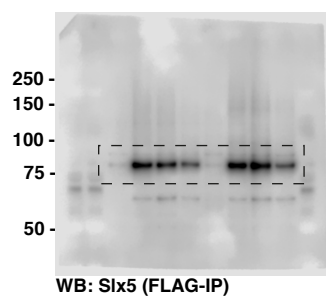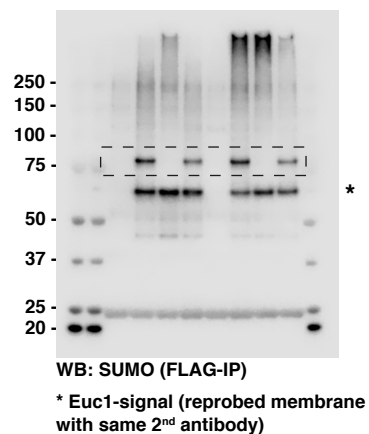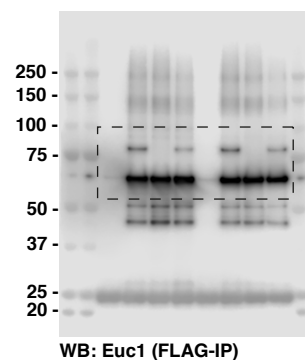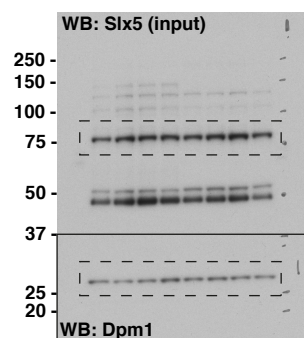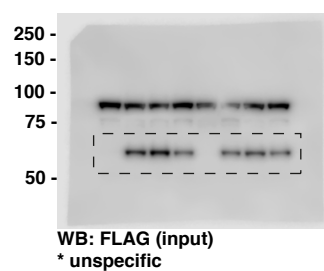

Source Data for Figure 5E.

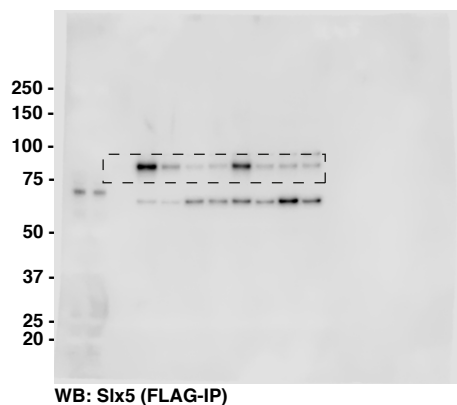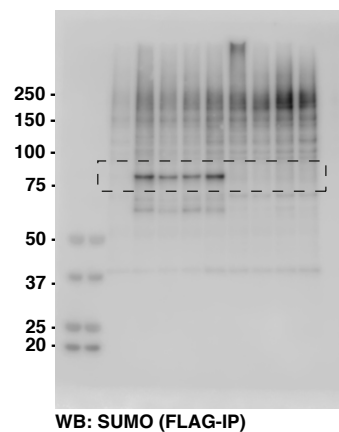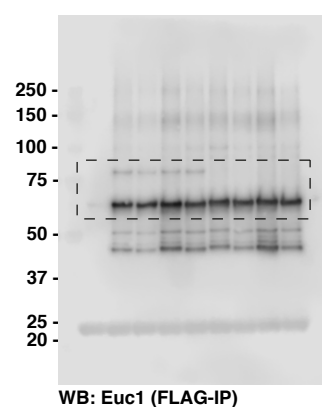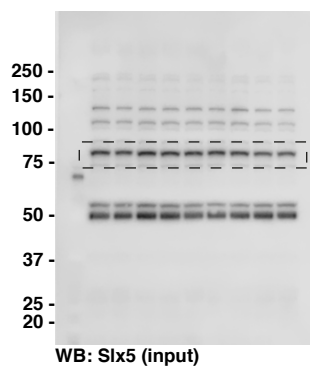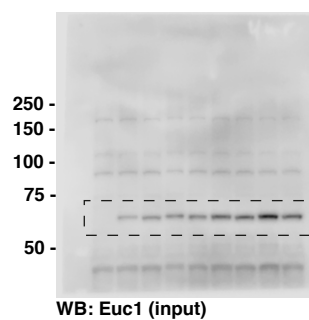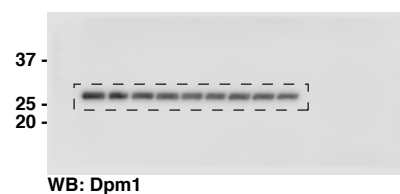

Supplement: Supplementary file 15 — Source Data for Figure 5 [file EMBJ-38-e100368-s013.pdf]

Source Data for Figure 6A.

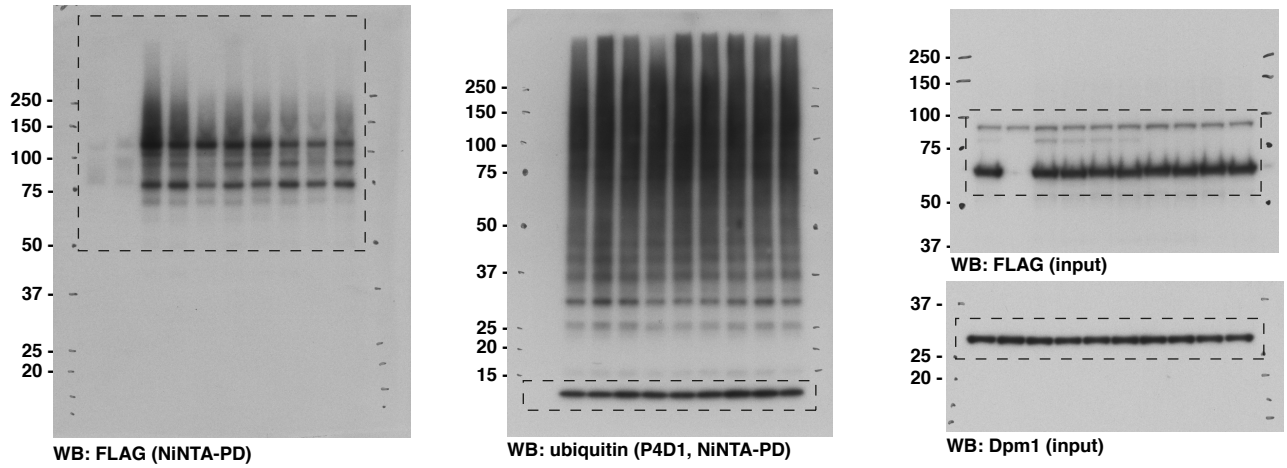

Source Data for Figure 6B.

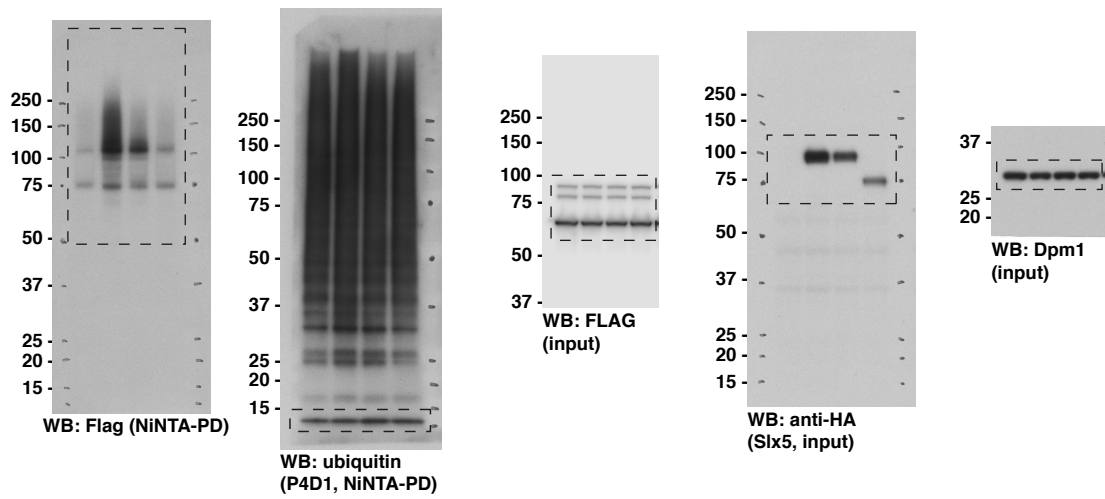

Supplement: Supplementary file 16 — Source Data for Figure 6 [file EMBJ-38-e100368-s014.pdf]
