# Supplementary material for: Slx5/Slx8‐dependent ubiquitin hotspots on chromatin contribute to stress tolerance
Source: EMBO J. 2019 Apr 23;38(11):e100368. doi: 10.15252/embj.2018100368 (PMC6545562; doi:10.15252/embj.2018100368)
Supplement: Supplementary file 12 — Source Data for Expanded View and Appendix [file EMBJ-38-e100368-s015.zip › embj2018100368-sup-0015-SDataAppendixFigs.zip!/EMBOJ-2018-100368R_SourceData_FigS1A.pdf]

Source data for Appendix Figure S1A

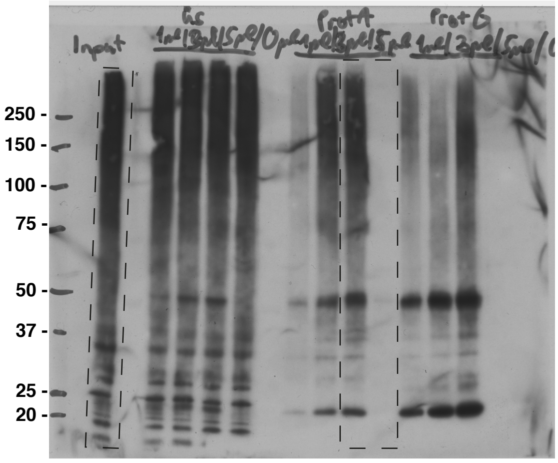

WB: ubiquitin (P4D1)
